# Synthesis, Optical Properties, and In Vivo Biodistribution Performance of Polymethine Cyanine Fluorophores

**DOI:** 10.1021/acsptsci.3c00101

**Published:** 2023-07-25

**Authors:** Md Shamim, Jason Dinh, Chengeng Yang, Shinsuke Nomura, Satoshi Kashiwagi, Homan Kang, Hak Soo Choi, Maged Henary

**Affiliations:** ^†^Department of Chemistry, ^‡^Center of Diagnostics and Therapeutics, Georgia State University, Atlanta, Georgia 30303, United States; § Gordon Center for Medical Imaging, Department of Radiology, Massachusetts General Hospital and Harvard Medical School, Boston, Massachusetts 02114, United States

**Keywords:** near-infrared dyes, *in silico* calculations, DFT study, photostability, *in vivo* biodistribution

## Abstract

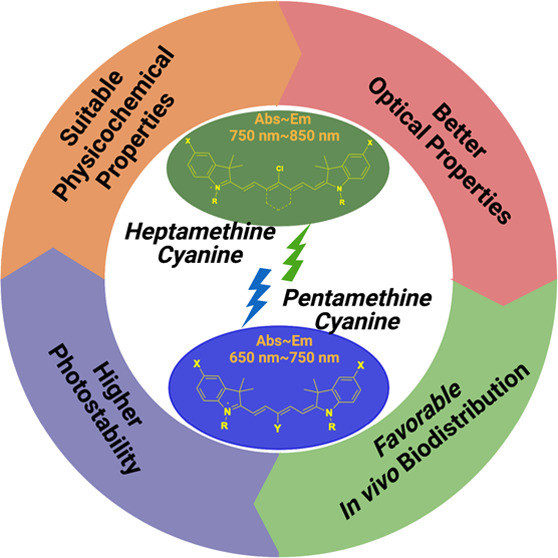

Near-infrared (NIR) cyanine dyes showed enhanced properties for biomedical imaging. A systematic modification within the cyanine skeleton has been made through a facile design and synthetic route for optimal bioimaging. Herein, we report the synthesis of 11 NIR cyanine fluorophores and an investigation of their physicochemical properties, optical characteristics, photostability, and *in vivo* performance. All synthesized fluorophores absorb and emit within 610–817 nm in various solvents. These dyes also showed high molar extinction coefficients ranging from 27,000 to 270,000 cm^–1^ M^–1^, quantum yields 0.01 to 0.33, and molecular brightness 208–79,664 cm^–1^ M^–1^ in the tested solvents. Photostability data demonstrate that all tested fluorophores **28**, **18**, **20**, **19**, **25**, and **24** are more photostable than the FDA-approved indocyanine green. In the biodistribution study, most compounds showed tissue-specific targeting to selectively accumulate in the adrenal glands, lymph nodes, or gallbladder while excreted to the hepatobiliary clearance route. Among the tested, compound **23** showed the best targetability to the bone marrow and lymph nodes. Since the safety of cyanine fluorophores is well established, rationally designed cyanine fluorophores established in the current study will expand an inventory of contrast agents for NIR imaging of not only normal tissues but also cancerous regions originating from these organs/tissues.

In the 21st century, with so many advancements in medical science, biomedical imaging modalities proved to be an effective tool for the detection of tumors, cellular structures, and functions.^1^ Despite the numerous research in this area, there are only six imaging modalities available to surgeons to identify cancerous cells, including X-ray, magnetic resonance imaging (MRI), ultrasound (US), computed tomography (CT), positron emission tomography (PET), and single-photon emission computed tomography (SPECT).^2^ However, none of these imaging modalities can be used in real-time imaging. Moreover, radiation associated with some of the imaging modalities (MRI, PET, CT, X-ray) still tied clinicians to depend on their naked eyes during intricate surgery.^3,4^ Therefore, high demand is growing for the real-time imaging modality, which will not only guide surgeons during complex and arduous surgery but also work as a safer process for both patients and healthcare providers.^5^ In that context, optical imaging in the near-infrared (NIR) region (650–1700 nm) has gained immense interest from clinicians as an attractive imaging modality to track biological targets.

Exogenous contrast agent plays a vital role in optical imaging.^4,6^ Important features for any organic dyes to use as contrast agents in optical imaging are absorption/emission profile, molar extinction coefficient, quantum yield, Stokes shift, and photochemical stability. NIR cyanine fluorophores with their abilities of deeper tissue penetration, high imaging resolution, noninvasive performance, low-level attenuation, real-time imaging result, and ability to produce high-quality images are rigorously investigated to use in intraoperative imaging.^7,8^ Due to their highly tunable structures and improved physicochemical and optical properties, bright fluorophores provide detailed information about the cells under *in vivo* study with consistent imaging.^9,10^ The optical properties and fundamental structures of various classes of fluorophores that may be utilized as contrast agents for *in vivo* optical imaging are displayed in Figure 1. Generally, cyanine fluorophores have two terminal nitrogen-containing heterocycles connected by a polymethine bridge. These heterocycles create electron delocalization across the polymethine chain through electron donor and acceptor capabilities.^11^ Based on the length of the polymethine chain, cyanine dyes are classified as trimethine cyanine (Cy3), pentamethine (Cy5), and heptamethine (Cy7).^12^

**Figure 1:**
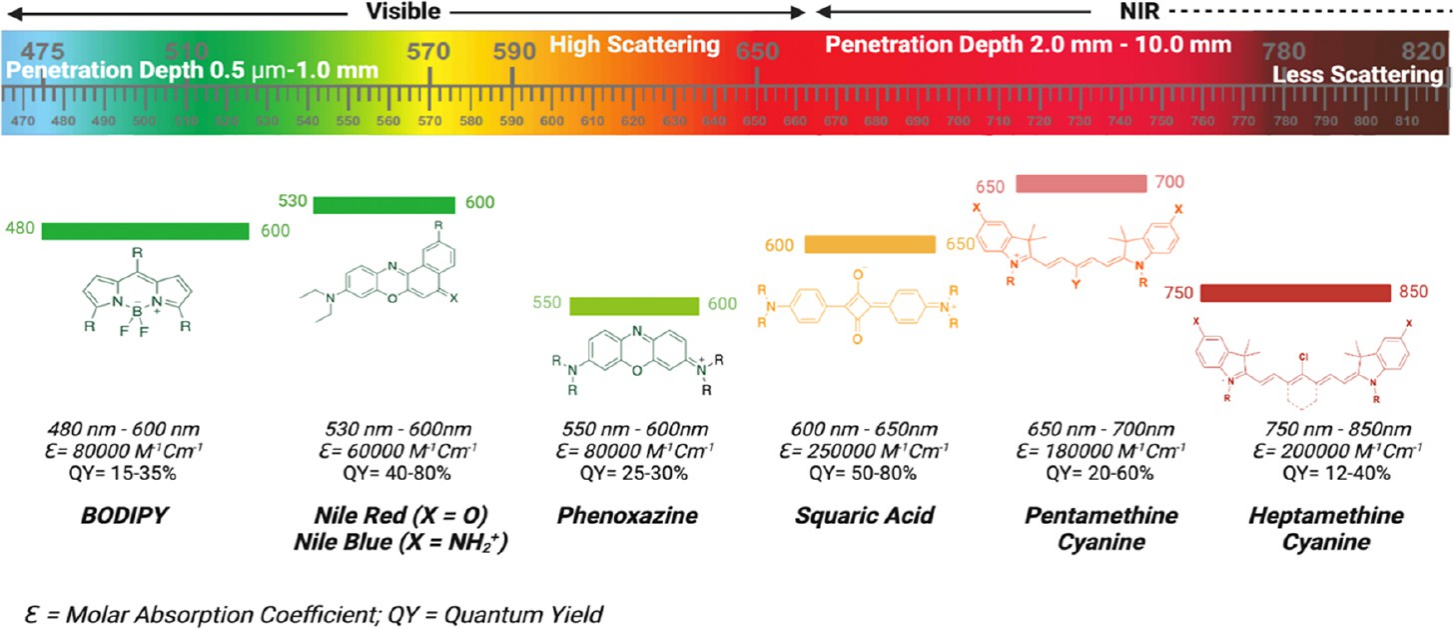
Core structures of various classes of fluorophores with their optical characteristics and absorption/emission profile considered as potential contrast agents for *in vivo* optical imaging.

*In vivo* biodistribution and clearance are strongly guided by the inherent properties of a fluorophore such as acid–base dissociation constant (p*K*_a_), molecular weight (MW), hydrogen bond donors and acceptors, total surface area, polarizability, distribution or partition coefficients, and photo and thermal stability. Our prior studies showed that *in vivo* distribution of hydrophobic pentamethine cyanine fluorophores is significantly controlled by physicochemical properties, specially the log D value,^13^ known as a distribution coefficient for any ionizable compounds, which measures the lipophilicity at certain pH. Generally, highly lipophilic molecules are less aqueous-soluble because of their poor solubility and low level of permeability. These characteristics increase the chance of off-center targeting and toxicity.^14,15^ However, several studies indicate that this range correlates with the increase of MW; log D > 1.7 is required for MW above 350 Da, log D > 3.1 is for MW above 400 Da, and log D > 4.5 is for compounds with MW above 500 Da.^7,16^

Appropriate biodistribution of any contrast agent is important to accumulate in the region or tissue of interest and reduce off-center targeting. High tunable features of cyanine fluorophores allow for possessing suitable physicochemical properties, which can lead to unique biodistribution patterns. However, the lack of proper optical signals from the contrast agents could result in misleading the biodistribution and clearance patterns.^17^ Well-defined optical properties with a high extinction coefficient, large Stokes shift, high quantum yield, and high photostability are needed to influence the *in vivo* performance.^18^ Besides, several approaches usually are taken to target a specific tissue of interest, by adding a targeting ligand to the cyanine molecule, without any targeting ligand, and activable targeting approach.^19^ Among those, one way is the “structure inherent targeting” approach.^20^ This concept uses the fluorophore without additional ligands (targeting and isolating) or additional structure complexity to reduce the overall complexity and toxicity during biodistribution. The underlying principle of the structure inherent targeting is that each fluorophore showed inherent pharmacophore properties owning to distinct chemical compositions that lead to a specific biodistribution pathway.

Flexible tunable features of cyanine fluorophores by adding or removing certain functional groups are advantageous for achieving target-selective imaging. Our group previously reported the correlation between structure and biodistribution patterns, which led to targeting various organs such as thyroid and parathyroid, cartilage, and bone-specific imaging.^13,21^ Recently, we reported that one of the heptamethine cyanine fluorophore **18**, which has a log D value of 8.64 at pH 7.4 showed ubiquitous tumor targetability in breast and lung cancer tumors with a high tumor-to-background ratio (TBR).^22^ Herein, we reported the synthesis of 11 pentamethine and heptamethine fluorophores, quantum computation, optical characteristics, photostability, and *in vivo* biodistribution performances for these fluorophores. The main objective of this research is to investigate the physicochemical, optical, and photostability properties of the selected cyanine fluorophores and show their effects on the biodistribution character. To the best of our knowledge, no scientific report has been made yet to correlate the effect of these properties combined on their biodistribution pattern.

## Results and Discussion

### Synthesis

**Scheme 1:**
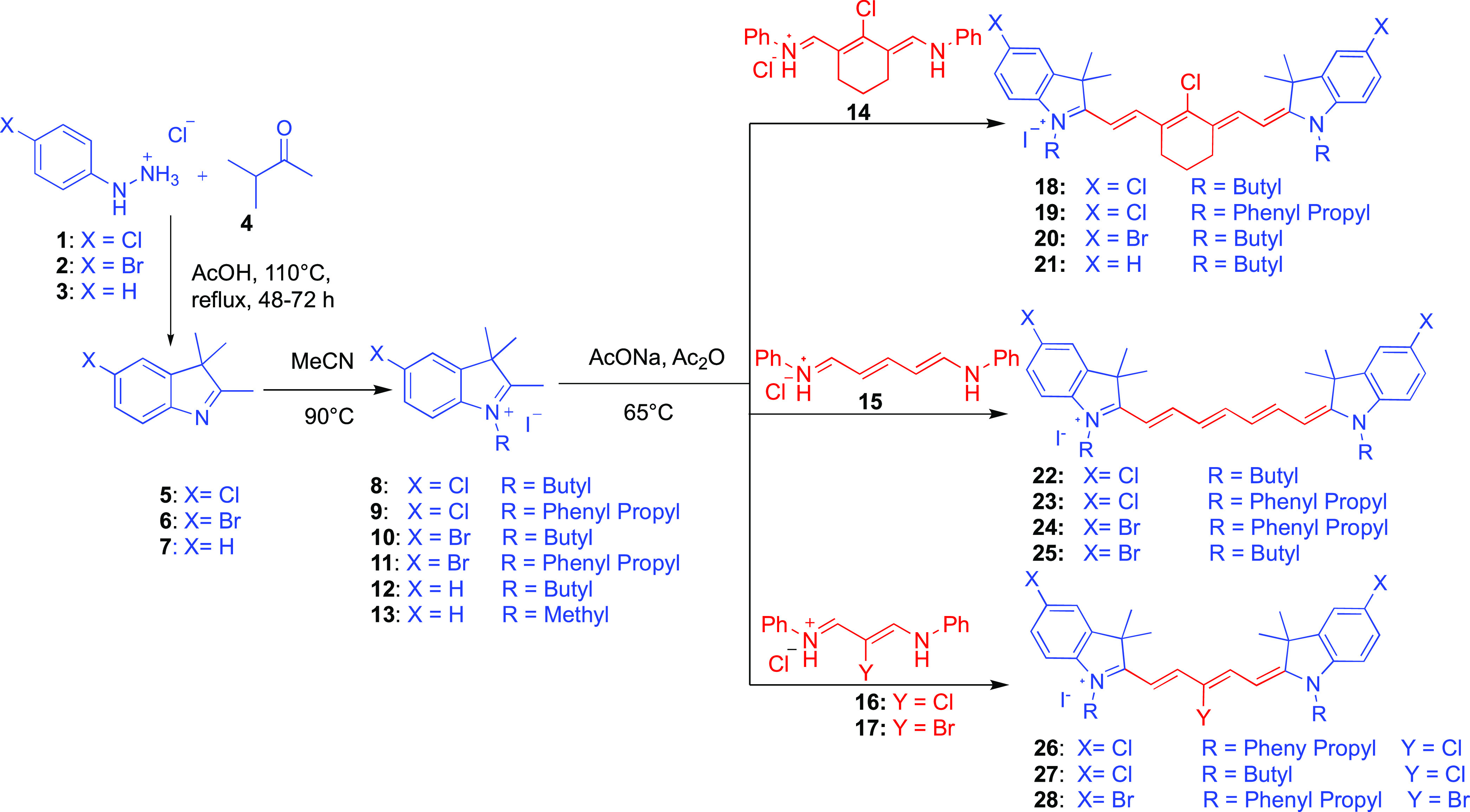
Synthetic Route for the Preparation of Cyanine Fluorophores **18–28**

As presented in Scheme 1, the synthesis started with the formation of indole rings **5–7** through Fischer indole synthesis. A starting material 4-substituted phenyl hydrazine derivative **1, 2**, or **3** was refluxed under an acidic condition with 3-methylbutan-2-one **4** for cyclization. Each cyclization reaction proceeded through the formation of imine derivatives and refluxed for 48–72 h at 110 °C to complete the reaction. After cooling the reaction mixture to room temperature, substituted indole rings **5–7** were achieved as a brown oil by extracting the reaction mixture in DCM and NaHCO_3_(aq). In the next step, heterocyclic 3*H*-indolium salts **8–13** were synthesized according to a previously reported procedure by our group,^10^ where *N-*alkylation to the cyclic indole rings was obtained by refluxing with various alkyl halides (Iodomethane, 1-Iodobutane, 1-bromo-3-phenyl propane) in boiling acetonitrile. The heterocyclic salts **8–13** were purified by performing several recrystallizations in DCM:ether, acetone:ether, EtOAc/ether, and MeOH/ether. After purification, each of these individual salts was allowed to be condensed with various linkers **14, 15, 16, or 17** separately under basic conditions to form the final desired products. For example, heptamethine cyanine derivatives containing cyclohexenyl rings **18–21** were achieved by the condensation reaction between Vilsmeier–Haack reagent^23^**14** and individual heterocyclic salt **8, 9, 10, 12**, or **13.** Another version of heptamethine cyanine fluorophores with an open chain polymethine bridge as seen in dyes **22–25** were accomplished by the condensation reaction between commercially available linker **15** and individual salt **8, 9, 11**, or **10.** These condensation reactions were performed under basic conditions between individual salt with linker **14** or **15** at a 2:1 ratio in boiling acetic anhydride for 4–6 h. For the pentamethine cyanine fluorophores **26–28**, the same condensation reaction conditions were maintained. For these products, individual heterocyclic salt **9, 8** or **11** was allowed to condense with polymethine chain **16** or **17**.^24^ Immediately after the reaction mixture was quenched, it was cooled down to room temperature. Finally, the crude products were purified by flush column chromatography in 5% methanol in DCM and dried under vacuum to obtain green crystals for the heptamethine cyanine fluorophores and blue crystals for the pentamethine cyanine fluorophores.

### Physicochemical Properties

*In silico* calculations of physicochemical properties, which included well-defined rotatable bonds, hydrogen bond donor–acceptor groups, hydrophobicity, and polar surface areas are crucial for rationally designing drug molecules. These properties can correlate the relationship between an optimized structure and its biological activities, such as permeability, retention, and clearance. Modifying the structures of any small molecules can alter these properties. For example, for an increased lipophilicity of a certain fluorophore at a certain pH, log D (distribution coefficient) predicts that molecules will partition faster into lipid cell membranes. The topological polar surface area (TPSA) predicts intestinal absorption.^25^ Higher TPSA means low permeability because of more retention in the vascular system, such as a larger molecular volume greater than 40 kDa can increase the residence time in the peripheral compartments.^26^ However, the low MW can increase the permeability. Therefore, to predict the localization of the fluorophores in a specific tissue, permeability, cellular uptake, retention, and clearance the physicochemical properties were examined by ChemAxon (JChem plugin).^27^ The results obtained from *in silico* calculations are presented in Table 1. Most molecular volumes of the synthesized fluorophores lied between 531 and 699 Å^3^, while their TPSA remained the same at 6.25 Å^2^. The log D values at pH 7.4 for the fluorophores **18–28** are fallen between 7.44 and 10.91, and the polarizability value changes from 64 to 88. Among the log D values presented in Table 1, fluorophore **21** showed the lowest hydrophobicity value, whereas fluorophore **19** showed the highest.

**Table 1:**
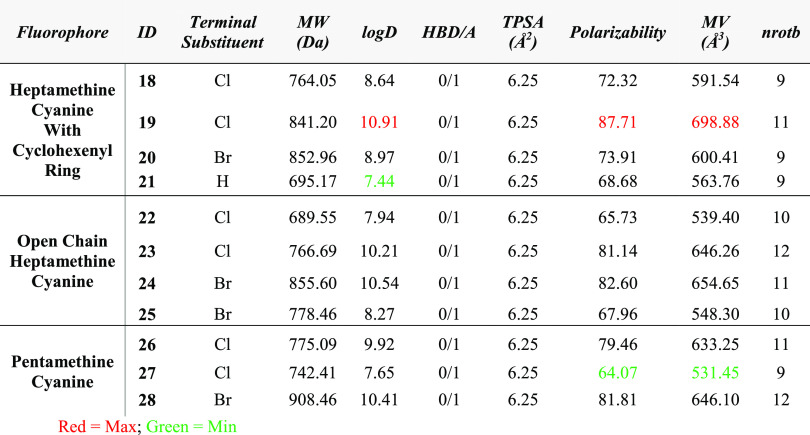
Physicochemical Properties of Fluorophores **18–28** Were Calculated by Using Marvin and JChem Calculator Plug-Ins (ChemAxon)^a^

a The data calculated (at pH 7.4) include log D, polarizability, number of rotatable bonds (nrotb), molecular volume (MV, Å^3^), topological polar surface area (TPSA, Å^2^), and molecular weight (MW, Da).

Our preliminary observation is that a large alkyl chain increases hydrophobicity. In addition to large alkyl chains (phenyl propyl), fluorophore **19** also has three Cl atoms in the structure and a cyclohexenyl ring in the middle, which are the reason behind the highest log D at pH 7.4. Although TPSA remains the same for all of the fluorophores, polarizability and molecular volume change with the structures. We observed a linear relationship between molecular volumes and polarizabilities. Polarizability increased with the increment of molecular volume. Fluorophore **19** is the most polarizable among the fluorophores with its largest molecular volume (698.88 Å^3^), while fluorophore **27** is the least polarizable with its lowest molecular volume (531.45 Å^3^).

### Optical Characterization and Explanation of Absorption and Emission Profiles of the Selected Fluorophores by DFT Calculations

*In vivo* success of any contrast agent highly depends on the absorption and emission profiles, molar extinction coefficient, quantum yield, molecular brightness, physicochemical properties, and photochemical stability.^24^ The synthesized fluorophores can be classified into two main groups, which are distinguishable based on their polymethine chain lengths: heptamethine cyanine fluorophores, ranging from **18** to **25**, and pentamethine cyanine fluorophores, specifically **26** to **28**. Different types of polymethine bridges have a substantial impact on the structural geometry of the fluorophores and the absorption and emission profiles. Initially, we tried to understand the substituent effect on optical properties in four different media. We measured optical properties in polar protic EtOH, polar aprotic DMSO, and two buffer solutions, phosphate-buffered saline (PBS, pH ∼ 7.4) and 4-(2-hydroxyethyl)-1-piperazine-ethanesulfonic acid (HEPES, pH ∼ 7.4). Organic solvents such as ethanol (EtOH) and dimethyl sulfoxide (DMSO) are often used in spectroscopic experiments to observe the absorption and emission maximum, whereas buffer solvents PBS and HEPES were used to mimic the biological environment, as these synthesized fluorophores are intended to be used in bioimaging. The absorbance profiles of these fluorophores were recorded at various concentrations (0.8, 1.6, 2.4, 3.2, 4.0 μM) and the absorbance values obtained were plotted against each of the concentrations. The measured media showed a linear correlation between absorbance and concentration, which is observed in Figures S1–S11, in accordance with the Beer–Lambert law. Measured absorption and emission profiles are summarized in Table 2, where heptamethine cyanine dyes **18–25** absorb around 649–815 nm and pentamethine cyanine dyes **26–28** absorb within 610–659 nm. The emission maxima for heptamethine cyanine fluorophores were also within around 668–817 nm, and pentamethine cyanine fluorophores were recorded in between 667 and 675 nm.

**Table 2:** Summary of Absorption and Emission Profiles of the Fluorophores **18–25** and **26–28** in Four Different Solvents; (I) EtOH, (II) DMSO, (III) HEPES, and (IV) PBS

	ID	EtOH		DMSO		HEPES		PBS	
fluorophore	#	λ_abs_	λ_em_	λ_abs_	λ_em_	λ_abs_	λ_em_	λ_abs_	λ_em_
heptamethine cyanine with cyclohexenyl ring	**18**	794	808	802	814	609	802	693	802
	**19**	795	811	804	817	753	777	750	779
	**20**	793	808	805	815	787	803	710	805
	**21**	783	803	799	811	778	796	775	800
open chain heptamethine cyanine	**22**	754	780	763	786	726	668	815	668
	**23**	760	783	761	793	649	671	668	674
	**24**	755	782	761	789	655	671	787	673
	**25**	756	783	761	790	753	777	751	775
pentamethine cyanine	**26**	657	672	659	675	615	671	622	675
	**27**	655	671	657	673	653	670	650	667
	**28**	656	671	657	673	608	672	610	673

The highest absorbance maximum was observed at 815 nm for open chain heptamethine cyanine **22** in PBS; however, heptamethine cyanine with cyclohexenyl ring **19** exhibited the highest emission wavelength at 817 nm in DMSO. We observed that the absorbance maxima red-shifted by 5–20 nm in DMSO but blue-shifted (20–130 nm) in buffer solutions. As reported, the emission spectra are highly influenced by the nature of the media, and large spectral shifts are observed in buffer solutions.^28^ Absorbance maxima (λ_max_) of heptamethine cyanine fluorophores **18–21** with cyclohexenyl rings were observed in the range of 661–805 nm, whereas open chain heptamethine fluorophores **22–25** were observed between 649–815 nm. However, peak broadening was observed for these fluorophores as they aggregate in buffer solutions. Among the tested fluorophores, most of the fluorophores **18, 23, 24, 26**, and **28** showed H-aggregates in both buffer solutions. In contrast, heptamethine cyanine dye **22** exhibited distinct aggregations when placed in different buffer solutions. Specifically, in PBS, J-aggregates were observed, causing a red shift in the absorption maximum to 815 nm. Meanwhile, in HEPES, H-aggregates were observed, which resulted in a blue shift of the absorption maximum to 726 nm. Different substituted groups, such as chlorine and bromine, have a negligible effect on the dye absorption and emission spectra. However, an extension of the pi system led to a red-shifted absorbance and fluorescence when comparing fluorophore **18** to fluorophore **26** (Table 2). Upon excitation at the absorbance maximum, 661 nm, the emission wavelength of fluorophore **18** in HEPES buffers was seen at 802 nm, which gave the largest Stokes shift, 140 nm, observed among these fluorophores.

**Figure 2:**
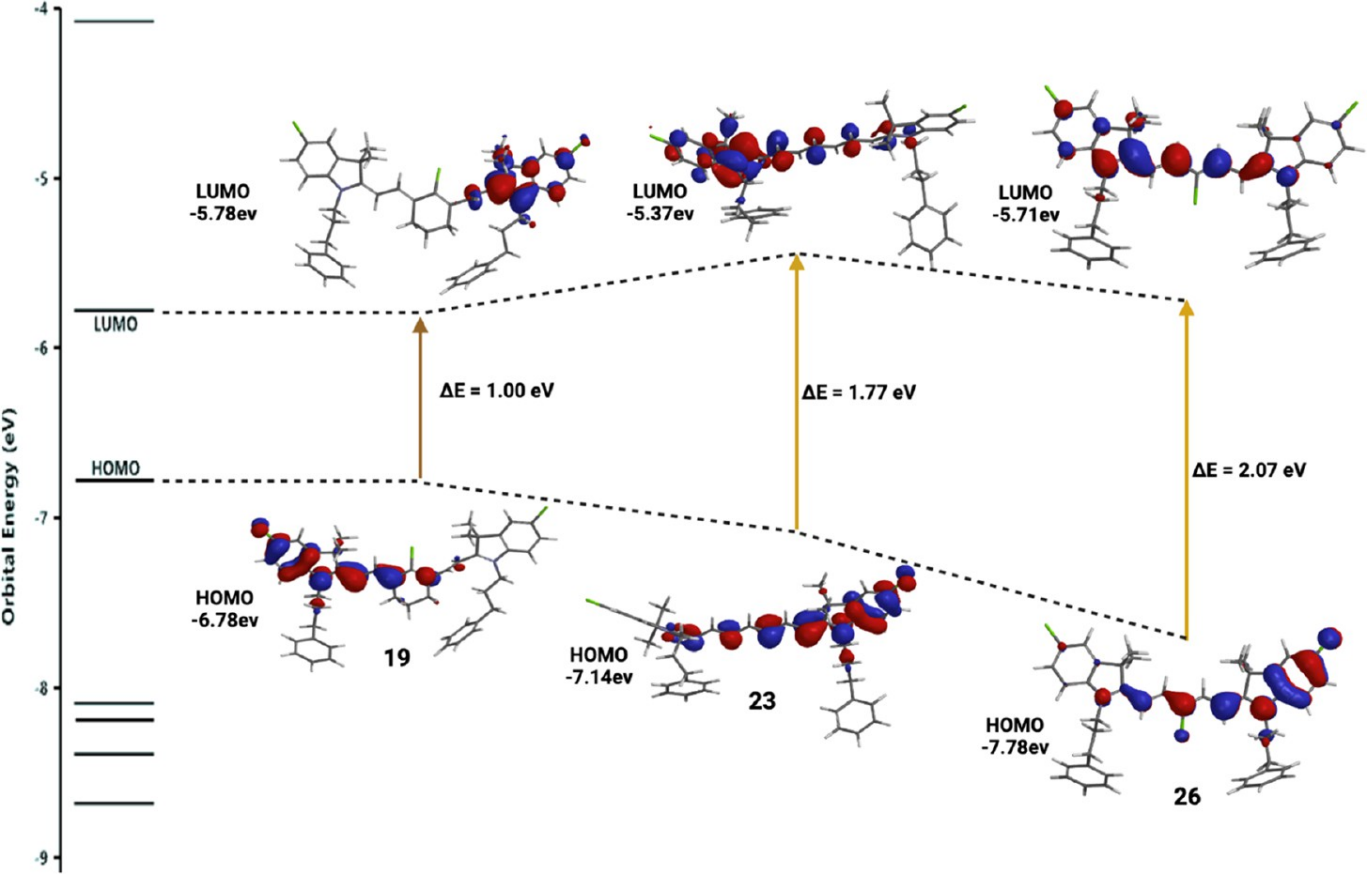
Theoretical calculations of frontier molecular orbitals for the optimized structures of cyanine **19**, **23**, and **26** based on DFT calculations at the B3LYP/6-311G(d,p) level.

To understand the contribution of the geometry and the pi system on the optical properties, quantum chemical calculations were performed on the selected fluorophores by frontier molecular orbitals (FMOs) in optimized structures based on the density functional theory (DFT) method with a 6-311G basis set. In comparison to pentamethine, the presence of one extra double bond conjugation in heptamethine fluorophores introduced more vibrational energy levels and decreased the energy difference between the ground states and the excited states. Moreover, the presence of a cyclohexenyl ring in the polymethine bridge enhances rigidity, made the overall structure flat, and decreased the energy band gap between FMOs.^29^Figure 2 shows the energy difference of optimized molecular structures between FMOs of fluorophores **19**, **23**, and **26**. The energy differences between the highest occupied molecular orbitals (HOMOs) and lowest unoccupied molecular orbitals (LUMOs) increased from 1.00 to 2.07 eV. These fluorophores have the same terminal indolium heterocycles but different polymethine chains such as fluorophore **19** with a cyclohexenyl ring in the polymethine bridge, fluorophore **23** with an open polymethine chain, and fluorophore **26** with one double bond shorter conjugation length polymethine chain. As reported, energy is inversely proportional to its wavelength. The wavelength for absorption and emission maxima decreased as following fluorophores **19**λ_abs_/λ_em_ > **23**λ_abs_/λ_em_ > **26**λ_abs_/λ_em_ with the increase of the energy difference from left to right as shown in Figure 2.

**Figure 3:**
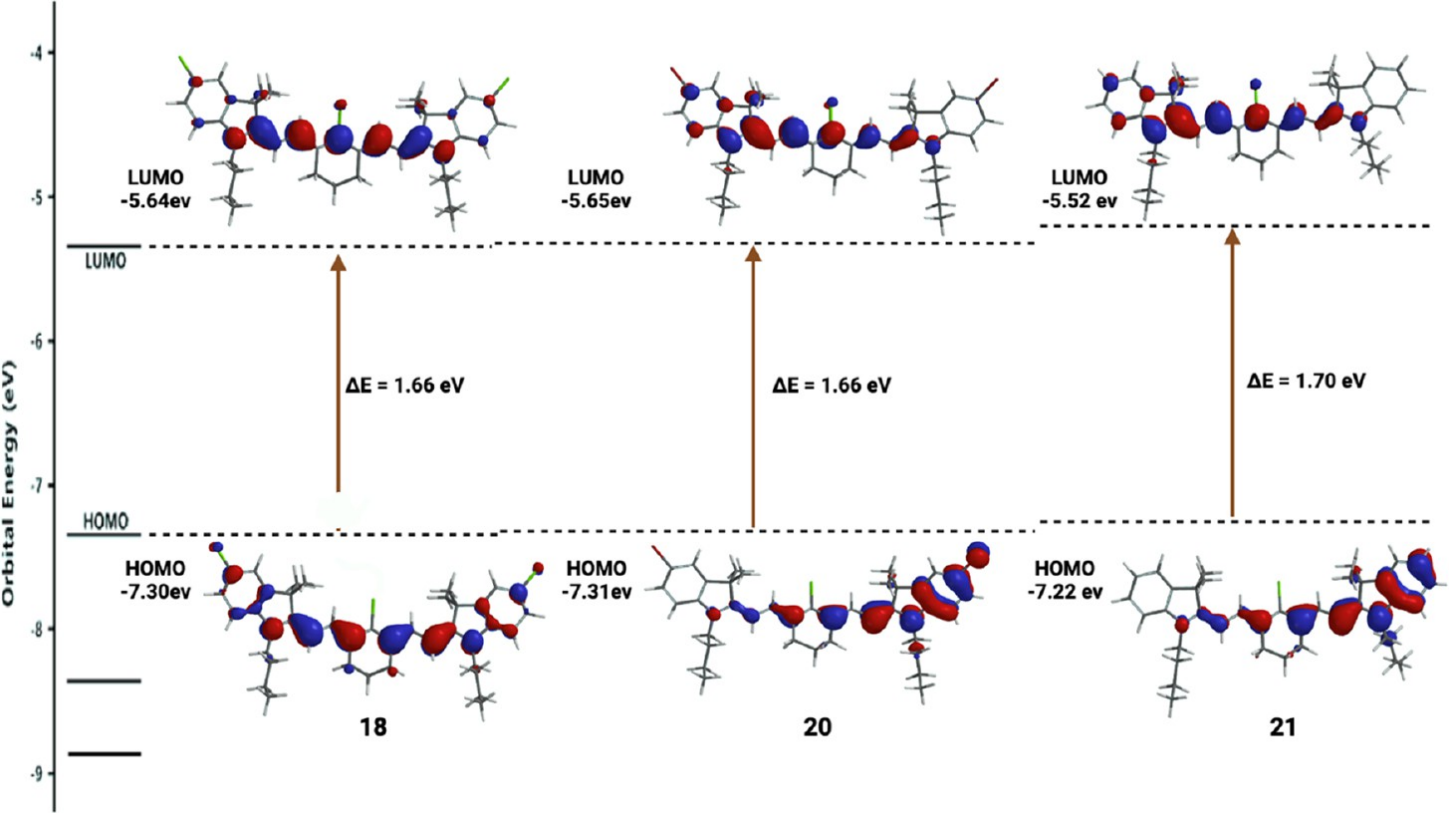
Theoretical calculations of frontier molecular orbitals for the optimized structures of fluorophores **18**, **20**, and **21** based on DFT calculations at the B3LYP/6-311G(d,p) level.

In addition, we performed DFT calculations on fluorophores **18, 20**, and **21** presented in Figure 3 to understand the substituent effect on the absorption and emission wavelengths. We observed that the substituents, such as the halogen atoms (Cl/Br), do not affect the energy gap, whereas the presence of a hydrogen atom changes the HOMO–LUMO gap slightly. Therefore, fluorophore **18**, having a substituent Cl atom in the heterocyclic rings, and fluorophore **20**, containing a Br atom in the heterocyclic rings, showed almost similar absorption and emission wavelengths. But fluorophore **21**, without any halogen substituent showed slightly less absorption and emission wavelengths. This outcome can be explained by the resonance and induction effect of the halogen atom. Halogen atoms in the heterocyclic rings possibly increase the electron density by pushing electrons to the conjugation system. On the other hand, it stabilized the conjugation of the polymethine bridge by withdrawing electrons through an inductive effect. This phenomenon pushes the overall HOMO and LUMO gap narrower. For that reason, halogen atoms substituents in the heterocyclic rings and in the polymethine bridge provide a little more stable structures with higher absorption and emission maxima than their substituent hydrogen atoms.

The molar extinction coefficient (Figures S12–S15) is an intrinsic property of any fluorophore and is important for high image resolution. Generally, fluorophores with high molar absorptivity and quantum yield showed high molecular brightness, which means that less dosing is required for *in vivo* applications. These properties vary with the structure change and are difficult to predict beforehand. The optical properties of the synthesized fluorophores were measured in various media. All of the data are summarized in Table 3. Synthesized fluorophores showed higher molar extinction coefficients and molecular brightness in organic solvents but lower extinction coefficients and brightness in buffer solutions. Due to their hydrophobic nature, all of the synthesized fluorophores tend to aggregate when in aqueous environments. This aggregation causes a reduction in their molar extinction coefficient and molecular brightness, ultimately affecting their ability to absorb and emit light. Furthermore, the aggregated state of the dye molecules can result in fluorescence quenching, which further diminishes their brightness.^30^ We observed molar extinction coefficients of fluorophore **18** in the decreasing order 270,000, 203,000, 44,000, and 41,000 cm^–1^ M^–1^ in EtOH, DMSO, HEPES, and PBS, respectively. We also found that fluorophore **18** showed significantly lower quantum yield and molecular brightness in buffer solutions. We observed that the molar extinction coefficient of fluorophore **18** decreased in the order of 270,000, 203,000, 44,000, and 41,000 cm^–1^ M^–1^ in EtOH, DMSO, HEPES, and PBS, respectively. Fluorophore **18** also exhibited significantly lower quantum yield and molecular brightness in buffer solutions. The highest molar extinction coefficient (270,000 cm^–1^ M^–1^) was observed for fluorophore **18**, while the highest molecular brightness (79,664 cm^–1^ M^–1^) was observed for fluorophore **21**, and the highest quantum yield (0.33) was observed for fluorophores **21** and **24** in EtOH. In contrast, the lowest molar extinction coefficient (27,000 cm^–1^ M^–1^) was observed for fluorophore **24**, the lowest molecular brightness (208 M^–1^ cm^–1^) was observed for fluorophore **22**, and the lowest quantum yield (0.01) was observed for fluorophores **20** and **21** in PBS. Furthermore, it was observed that the heptamethine cyanine dyes (**18–22**) had higher extinction coefficients in organic solvents than pentamethine cyanine dyes (**26–28**).

**Table 3:**
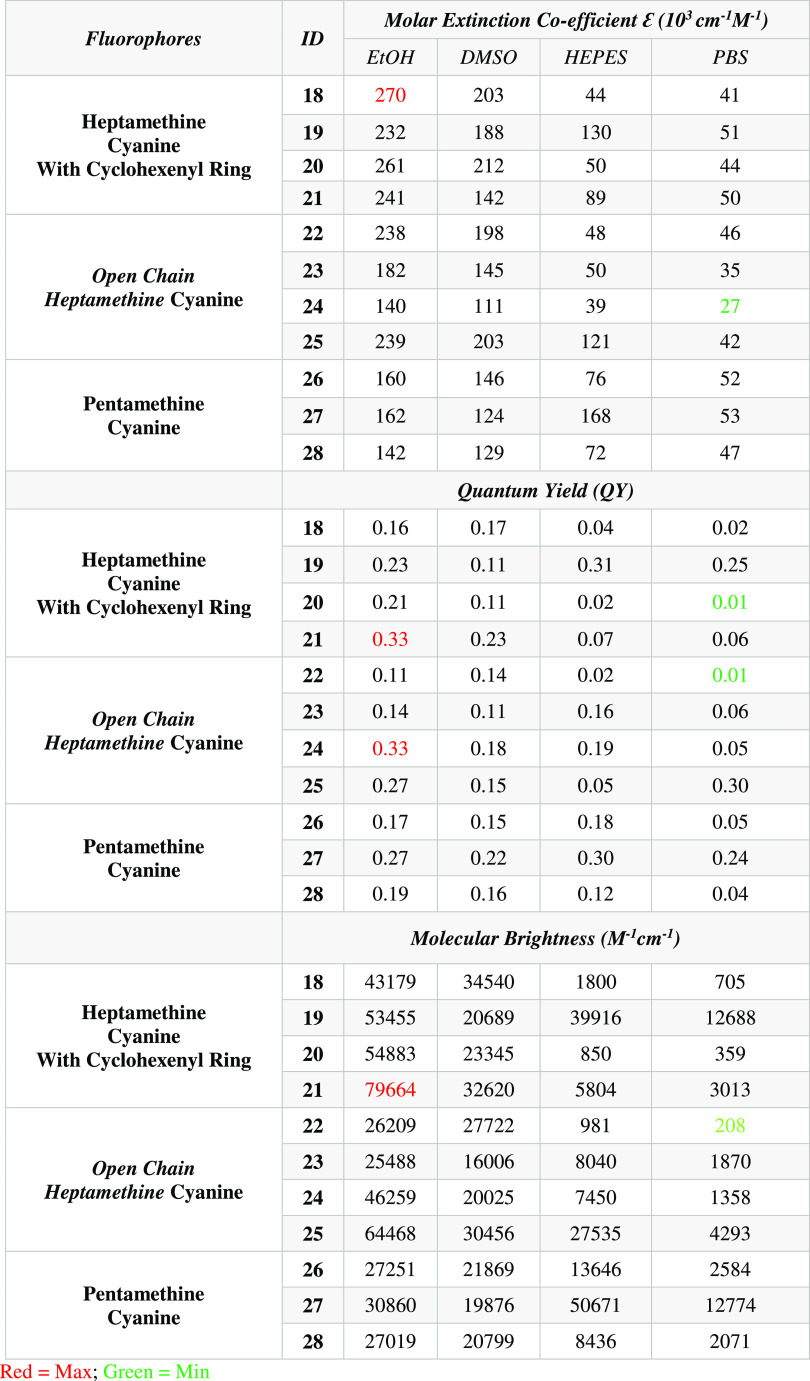
Summarized Optical Properties of Fluorophores **18–28**^a^

a All measurements were performed in 2 different organic solvents (EtOH and DMSO) and 2 different buffer (HEPES and PBS), and ICG was used as a reference (Φ_ICG_ = 0.043).

### Photostability Study

**Figure 4:**
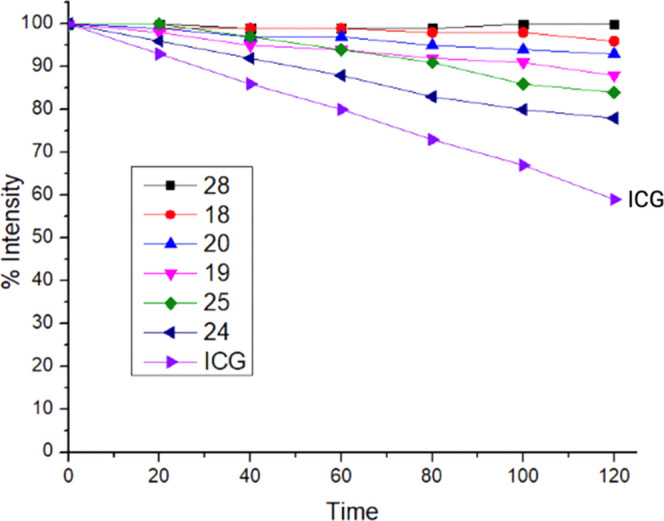
Photostability data of the selected fluorophores **18, 19, 20, 24**, **25**, and **28** compared against commercially available FDA-approved heptamethine cyanine dye **ICG**. All of the data were collected in every 20 min intervals under continuous irradiation with a xenon lamp at 150 W for 2 h and presented based on the % reduced fluorescence intensity starting from 100%.

In consideration of theranostic (therapeutics and diagnostic) applications, NIR cyanine fluorophores are synthesized for fluorescence imaging, photodynamic therapy (PDT),^31^ photothermal therapy (PTT),^32^ optoacoustic imaging,^33^ and cancer immunotherapy.^22^ One of the approaches before using these fluorophores in those applications is to study their biodistribution and clearance pattern *in vivo*. Generally, after intravenous injection of these contrast agents, 4–8 h is required to localize in the targeted area and clear from other background tissues and organs.^29,34^ Organic fluorophores used as contrast agents in those processes are light-sensitive, and prolonged exposure to light can induce photodegradation. Photobleaching is an outcome of the photodegradation process, which may cause unwanted toxicity and harmful effects. Also, it can limit the overall optical applications of the fluorophores during the *in vivo* process. Photobleaching is typically observed in the long-wavelength cyanine fluorophores because of their long polymethine bridge when they are in solution.^35^ Besides, it is important to know about the handling and storage protocols of these fluorophores. Therefore, we performed photostability studies on selected fluorophores **18, 19, 20, 24, 25**, and **28** versus the FDA-approved indocyanine green (ICG) by continuous irradiation with a xenon lamp at 150 W for 2 h. The power density of 150 W is much higher than the required power for fluorescence imaging, considering that the time it takes for a fluorophore to clear from the background and accumulate in sufficient quantities in the targeted tissues is unknown. However, using high-power sources can be justified if the fluorophore remains stable and demonstrates better photostability than ICG under these conditions. This is because a fluorophore, which showed better photostability by enduring a high light source would exhibit less photobleaching if there is a low energy source throughout the process. By considering this, an aliquot of the stock solution (1 mM in DMSO) was diluted in ethanol, and the fluorescence intensity was measured at the excitation wavelength same as their absorption maxima (nm). Photodegradation rates were measured every 20 min intervals for the selected fluorophores based on the % reduced fluorescence intensity starting from 100%. We observed that in dark conditions at room temperature, the selected fluorophores showed no photobleaching for over 24 h, while in the presence of light, all of the fluorophores showed photodegradation. Obtained results are presented in Table S16 and Figure 4. All of the tested fluorophores showed better stability than ICG possibly due to their rigid structure with fewer rotatable bonds, which reduces their susceptibility to chemical and photodegradation. In contrast, ICG has a more flexible structure with more rotatable bonds (14 nrotb), making it more prone to degradation over time under exposure to light.^36^ Fluorophores **18** and **28** degraded 1–4%, fluorophores **20** and **19** degraded 7–12%, and fluorophores **24** and **25** degraded 16–22%, while ICG degraded 41% at the end of 2 h. The photostability of the fluorophores decreases in the order **28** > **18** > **20** > **19** > **25** > **24** > **ICG**, which demonstrates that these dyes with short polymethine bridge fluorophore **28** and the presence of cyclohexenyl rings in fluorophores **18**, **19**, and **20** in the polymethine bridge showed increased photostability. Among the tested fluorophores having terminal heterocyclic indolium rings with a phenyl propyl group and polymethine bridge without the cyclohexenyl ring in the middle **24** showed significant photobleaching than its counterparts.

### Biodistribution Study on Synthesized Compounds 18–28

**Figure 5:**
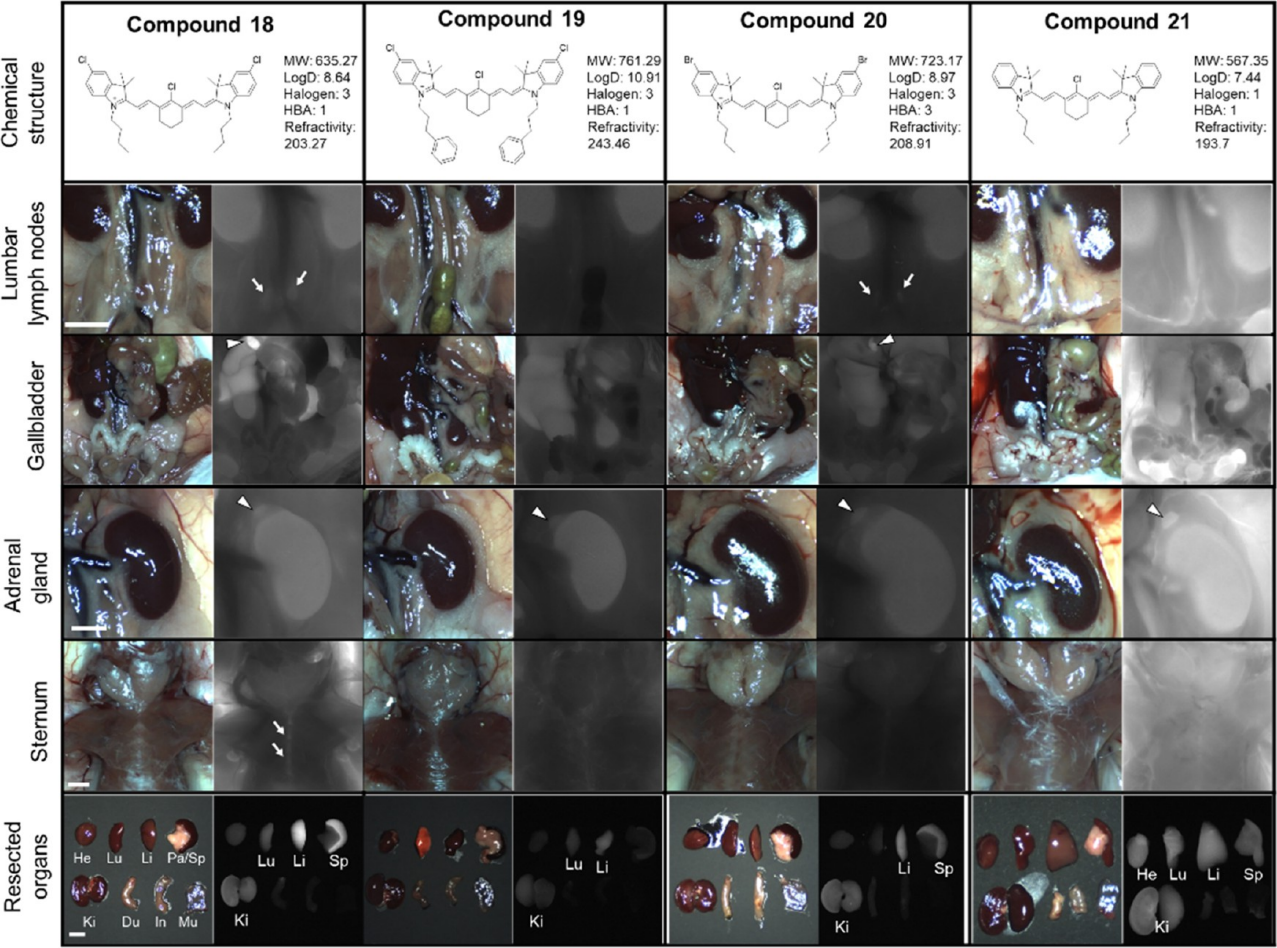
Targeting and biodistribution of heptamethine NIR fluorophores **18–21.** 25 nmol of each fluorophore was injected intravenously into CD-1 mice 4 h prior to imaging and resection. Abbreviations used are Du, duodenum; He, heart; In, intestine; Ki, kidney; Li, liver; Lu, lung; Mu, muscle; Pa, pancreas; and Sp, spleen. Arrows and arrowheads indicate the targeted organs.

**Figure 6:**
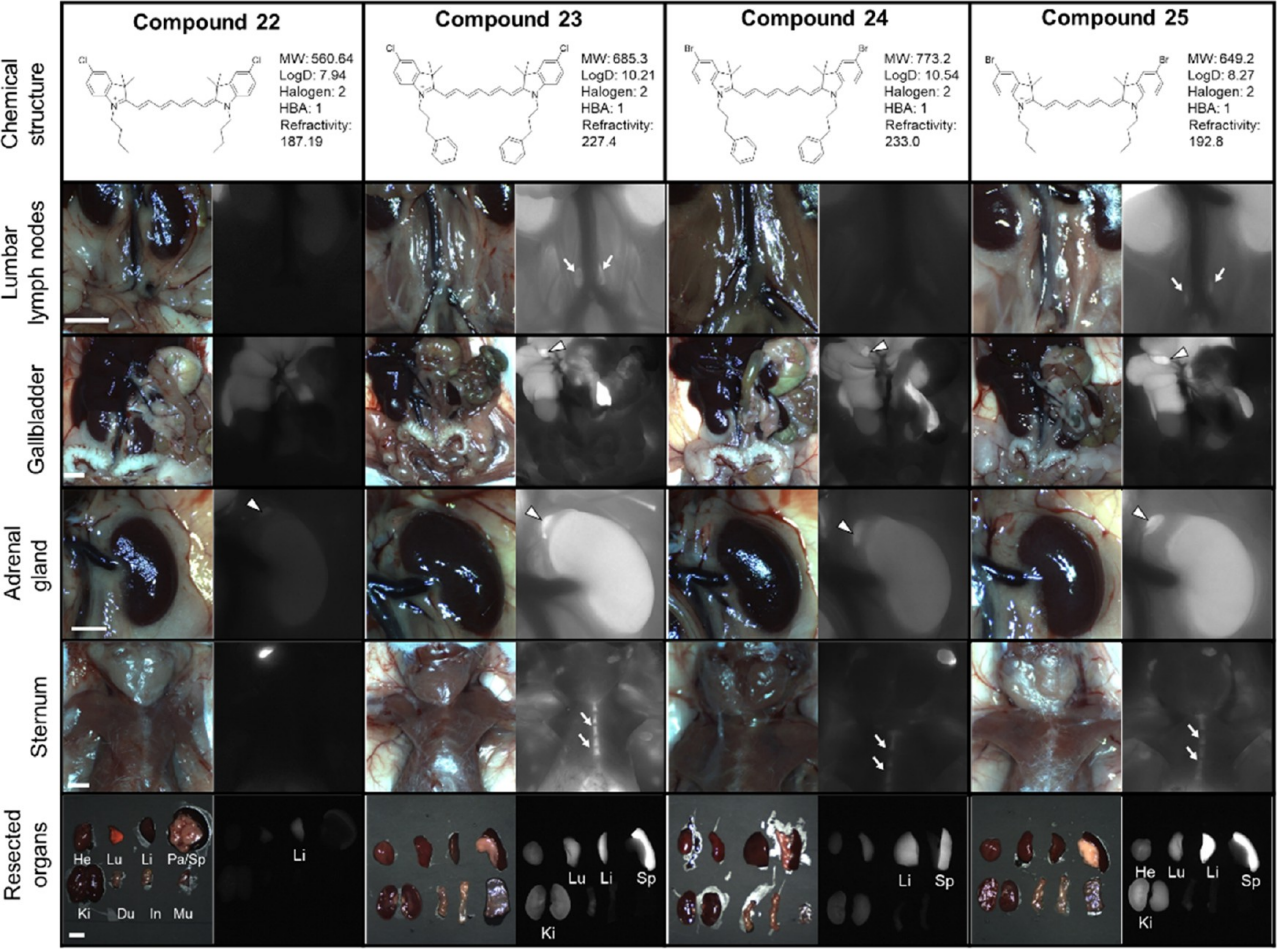
Targeting and biodistribution of heptamethine NIR fluorophores **22–25.** 25 nmol of each fluorophore was injected intravenously into CD-1 mice 4 h prior to imaging and resection. Abbreviations used are Du, duodenum; He, heart; In, intestine; Ki, kidney; Li, liver; Lu, lung; Mu, muscle; Pa, pancreas; and Sp, spleen. Arrows and arrowheads indicate the targeted organs.

**Figure 7:**
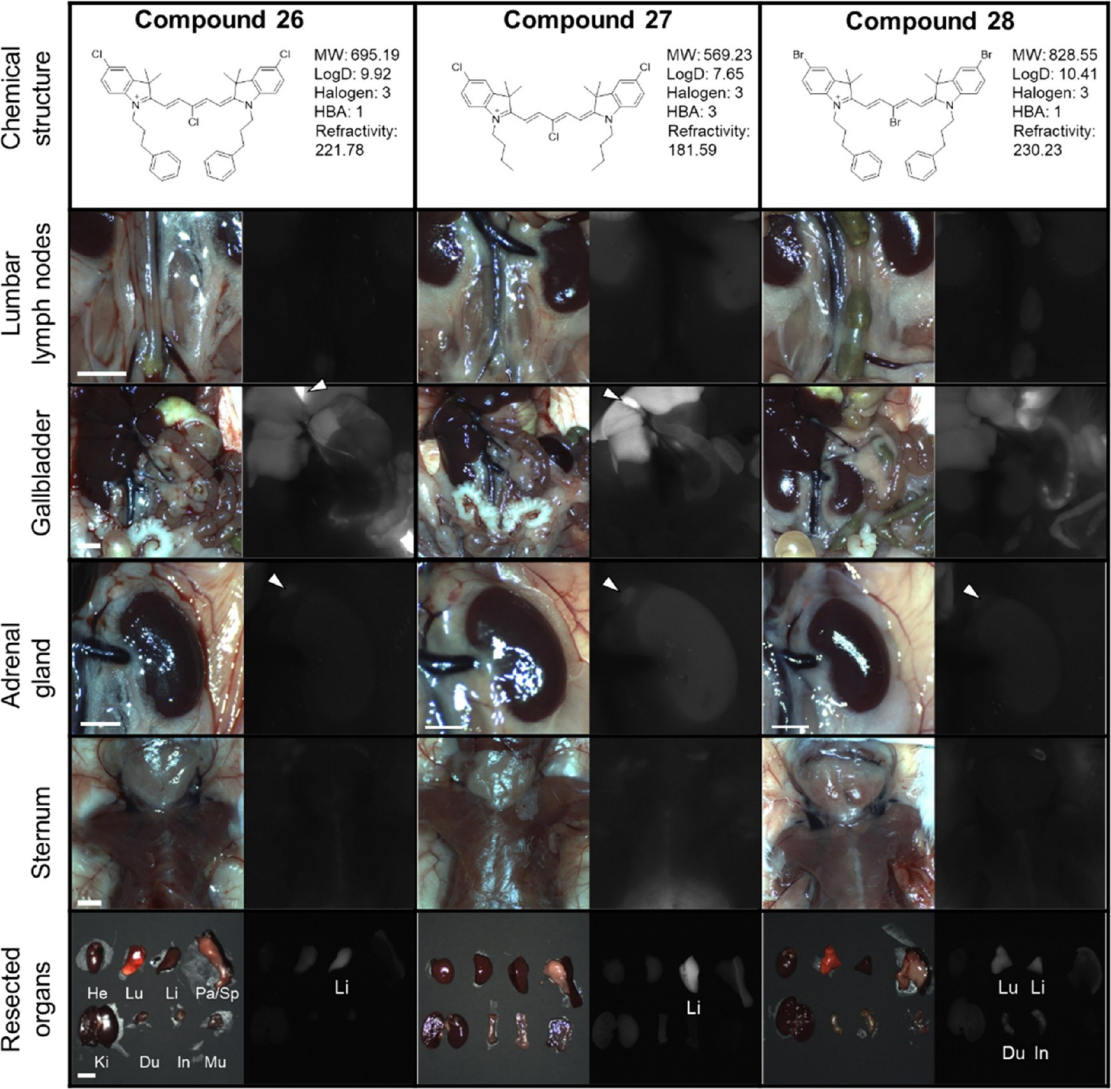
Targeting and biodistribution of pentamethine NIR fluorophores **26–28.** 25 nmol of each fluorophore was injected intravenously into CD-1 mice 4 h prior to imaging and resection. Abbreviations used are Du, duodenum; He, heart; In, intestine; Ki, kidney; Li, liver; Lu, lung; Mu, muscle; Pa, pancreas; Sp, spleen. Arrows and arrowheads indicate the targeted organs.

Newly synthesized fluorophores **18**–**28** were injected into CD-1 male mice to study their biodistribution and tissue/organ-targeting characteristics. 4 h prior to sequential intraoperative imaging (Figures 5–7, Table 4), 25 nmol of each fluorophore was injected intravenously. As shown in Figure 5, heptamethine fluorophores **18** and **20**, containing butyl chains on nitrogen atoms of heterocyclic backbone exhibited high signals in the primary and secondary lymphoid tissues including the bone marrow, spleen, lymph nodes, liver, gallbladder, and adrenal glands. The liver and gallbladder signals were consistent with hepatobiliary clearance. Chlorine-substituted fluorophore **18** produced higher signals in the bone marrow, spleen, and lymph nodes compared to bromine-substituted fluorophore **20**. However, fluorophore **21**, without halogens on the sides, showed high background signals due to the serum protein binding during systemic circulation.^22^ On the other hand, as shown in Figure 6, fluorophore **22** has a similar structure to fluorophore **18**, without a cyclohexenyl ring and a meso-chlorine, and showed reduced signals in the bone marrow, lymph node, adrenal gland, liver, and gallbladder compared to fluorophore **18**. Interestingly, lipophilic heptamethine fluorophores **23**, **24**, and **25** without a central cyclohexenyl ring and a meso-chlorine atom had high signals in the sternum bone marrow, gallbladder, and spleen, with improved targetability to the adrenal glands. Among these, fluorophore **23** with a terminal chlorine substituent and *N-*phenyl propyl showed the highest signal intensity in the bone marrow, spleen, and lymph nodes compared to other fluorophores, including those with bromine and *N*-butyl chain substitution. Interestingly, the pentamethine-based structures **26**, **27**, and **28**, such as halogen substituents and the *N*-butyl or *N*-phenyl propyl chain, provided high uptake in the liver and rapid hepatobiliary clearance with decreased signals in the bone marrow, adrenal gland, and lymph nodes due to the increased refractivity compared with heptamethine cyanines (Figure 7).

**Table 4:** Targeting Properties and Biodistribution of NIR-Emitting Fluorophores **18–28** at 4 h Post-Injection^a^

ID	AG	LN	Ga	BM	He	Lu	Li	Pa	Sp	Ki	Du	In
**18**	+++	+++	+++	++	++	+++	+++	++	+++	+++	+	+
**19**	++	+	+++	+	+	++	+++	+	++	++	–	–
**20**	+++	++	+++	+	++	+	+++	+	+++	+++	+	–
**21**	+++	+++	+++	++	+++	+++	+++	+++	+++	+++	+	–
**22**	+	–	+++	–	–	++	+++	–	++	+	–	–
**23**	+++	+++	+++	+++	+++	+++	+++	+++	+++	+++	+++	+
**24**	+++	++	+++	+++	+++	+++	+++	+++	+++	+++	+	+
**25**	+++	++	+++	+++	+++	+++	+++	++	+++	+++	–	–
**26**	+	–	+++	+	–	++	+++	–	+	–	–	+
**27**	++	–	+++	+	+	++	+++	+	++	++	+	–
**28**	+	–	+++	+	–	+++	+++	–	+	+	+	++

a Abbreviations used are AG, adrenal gland; BM, bone marrow; Du, duodenum; Ga, gallbladder; He, heart; In, intestine; Ki, kidney; Li, liver; Lu, lung; LN, lymph node; Pa, pancreas; and Sp, spleen. Arrows and arrowheads indicate the targeted organs. The SBR of each organ/tissue relative to the muscle was quantified and labeled as −, 1 to 2; +, 2 to 3; ++, 3 to 5; and +++, > 5.

Table 4 shows the summary of targeting properties and biodistribution of heptamethine (**18–25**) and pentamethine (**27–28**) fluorophores. Overall, the meso-chlorinated heptamethine fluorophore **21** showed higher background tissue uptake with circulation in the bloodstream for a longer period of time compared to other substitutions due to the rapid binding to serum proteins.^22^ Pentamethine cyanines (**26–28**) are smaller than heptamethines and more susceptible to MW and log D values, thus excreting faster than larger lipophilic heptamethines (**23–25**).^13^

## Conclusions

In a search of suitable contrast agents for fluorescence imaging in the NIR window I region, eight heptamethine cyanine fluorophores **18–25** and three pentamethine cyanine fluorophores **26–28** were synthesized successfully with relatively high yields. We examined their physicochemical properties including well-defined rotatable bonds, hydrogen bond donor–acceptor groups, hydrophobicity, and polar surface areas. The calculated log D values at pH 7.4 for the fluorophores **18–28** are greater than 7.0, their polarizability values change from 64 to 88, and their molecular volumes are between 531 and 699 Å^3^, while their TPSA remained the same at 6.25 Å^2^. All presented fluorophores showed a high molar extinction coefficient and high quantum yield, and heptamethine cyanine fluorophores with a cyclohexenyl ring showed significantly higher extinction coefficients than other synthesized fluorophores. DFT calculations showed that the cyclohexenyl ring in the polymethine bridge enhances rigidity, made the overall structure flat, and decreased the energy band gap between frontier molecular orbitals (FMOs). We also observed that selected fluorophores **18, 19, 20, 24, 25**, and **28** showed way better photostability than FDA-approved heptamethine cyanine dye **ICG**. Due to the relatively high log D values (>7.0 at pH 7.4), most of the synthesized compounds showed strong signals in the bone marrow, gallbladder, adrenal gland, and lymph node with hepatobiliary clearance. Among the tested, hydrophobic fluorophore **23** targets the bone marrow and lymph nodes within 4 h post-injection upon intravenous injection, showing that changes in physicochemical properties significantly affect the biodistribution of these fluorophores via cellular uptake and clearance. Interestingly, pentamethine fluorophores **26**–**28** provided rapid hepatobiliary clearance with decreased signals in the secondary lymphoid tissues, despite the substitution of a halogen and an *N*-butyl or *N*-phenyl propyl chain due to the increased refractivity compared with heptamethine cyanines.

## Experimental Section

### Materials

All chemicals and solvents used in the synthesis and purification were of American Chemical Society (ACS) grade or HPLC grade. These chemicals were purchased from Fisher Scientific (Pittsburgh, PA), Sigma-Aldrich (Saint Louis, MO), Combi-Blocks (San Diego, CA), and Acros Organics (Pittsburgh, PA). TLC plates have silica gel 60 F_254_ (Merck EMD Millipore, Darmstadt, Germany) used to guide the completion of the reaction. A flash column chromatographic technique was used to purify the final fluorophores by using 60–200u, 60A classic column silica gel (Dynamic Adsorbents, Norcross, GA). Both ^1^H NMR and ^13^C NMR spectra were obtained using high-quality Kontes NMR tubes (Kimble Chase, Vineland, NJ) rated to 500 MHz and were recorded on a Bruker Avance (400 MHz) spectrometer using chloroform-*d* or DMSO-*d*_6_ containing tetramethylsilane (TMS) as an internal calibration standard set to 0.0 ppm. All chemical shifts were recorded in parts per million (ppm). Signals are labeled as follows: s = singlet, d = doublet, t = triplet, m = multiplet, dd = doublet doublets, br = broad, and coupling constants (*J*) are measured in hertz (Hz) throughout the experimental section. UV–vis/NIR absorption spectra were recorded on a Varian Cary 50 spectrophotometer. The melting points were determined with a Mel-temp melting point apparatus and were corrected.

### Optical Study, Photostability, Physicochemical Properties, and DFT Calculation

1 mM stock solution in DMSO of the synthesized fluorophores was prepared before starting any spectral measurement. Optical properties for the synthesized fluorophores were measured in four different solvents (EtOH, DMSO, HEPES, and PBS). Absorbance spectra were measured in a Varian Cary 50 absorbance spectrophotometer (190–1100 nm). Fluorescence emission and photostability were measured and emission spectra were recorded on a Shimadzu RF-5301PC spectrofluorometer. Physicochemical properties (Log D, molecular mass, TPSA, H bond D/A, polarizability, molecular volume, and rotatable bonds) were predicted by ChemAxon (JChem plugin). Theoretical calculations of frontier molecular orbitals (FMOs) for the optimized structures of selected fluorophores were calculated based on DFT calculations at the B3LYP/6-311G(d,p) level.

### *In Vivo* Biodistribution Study of NIR Fluorophores (Compounds **18–28**)

Under the supervision of the MGH IACUC, animals were housed in an AAALAC-certified facility and studied according to institutional protocol #2016N000136. In preparation for injection, CD-1 mice (25–30 g, 6 weeks, Charles River Laboratories, Wilmington, MA) were anesthetized with isoflurane and oxygen. A 10 mM stock solution of NIR fluorophores was prepared in DMSO, and 25 nmol of each molecule was diluted in BSA-containing saline (5 wt/v%) for intraoperative imaging. After 4 h post-injection, mice were imaged. For each experiment, camera exposure time and image normalization were held constant.

### Quantitative Analysis

The fluorescence and background intensity of a region of interest (ROI) over each tissue was quantified using ImageJ software (NIH, Bethesda, MD) version 1.45q. The signal-to-background ratio (SBR) was calculated as SBR = fluorescence/background, where background is the signal intensity of neighboring tissues such as muscle. All NIR fluorescence images for a particular fluorophore were normalized identically for all conditions of an experiment.

### General Procedure for the Preparation of Fluorophores **18–28**

Our research group previously reported the synthetic procedure of the precursors of the cyanine fluorophores.^13,19,22,37^ Individual heterocycle indolenium salts **8–13** (2 mol equiv) were taken in a clean dried round bottom flask (100 mL). These salts were then dissolved in acetic anhydride (5 mL) with Vilsmeier–Haack reagent **14** (1 mol equiv), commercially available glutaconaldehydedianil hydrochloride **15** (1 mol equiv), *N*-((1E,2Z)-2-chloro-3-(phenylamino)allylidene)benzenaminium **16** (1 mol equiv), or *N*-((1E,2Z)-2-bromo-3-(phenylamino) allylidene) benzenaminium **17** (1 mol equiv) in the presence of acetic anhydride (5 mL) and sodium acetate (3.5 mol equiv) to yield the cyanine fluorphores **18–28**, respectively. The reaction mixture was vigorously stirred for 2–8 h at 65 °C. During this period, the solution gradually turns dark green for the heptamethine cyanine fluorophores and dark blue for the pentamethine cyanine fluorophores. The reaction mixtures were monitored by vis/NIR spectrophotometry via analyzing the change of the relative ratios between the expected absorption band (> 750 nm for fluorophores **18–21**, **22–25**, >650 nm for fluorophores **26–28**) and the starting material absorption peaks (<500 nm) in methanol and the synthesis, followed by using thin layer chromatography (TLC) in dichloromethane (DCM) and 5% methanol as the eluting solvent. The individual reaction mixture was allowed to cool down to room temperature, and then the solid of each dye is collected and purified *via* flash column chromatography using 5–10% methanol in DCM. The solution of the pure fractions was collected and condensed under reduced pressure to produce dark green solids, which are then dried under a vacuum. All of the synthesized fluorophores were characterized and confirmed by ^1^H NMR and ^13^C NMR. The final fluorophores were determined to be >95% pure with fair to very good yields (41–72%).

#### 1-Butyl-2-((*E*)-2-((*E*)-3-(2-((*E*)-1-butyl-5-chloro-3,3-dimethylindolin-2-ylidene)ethylidene)-2-chlorocyclohex-1-en-1-yl)vinyl)-5-chloro-3,3-dimethyl-3H-indol-1-ium **18**

Yield (59%, 0.18 g); mp 156–158 °C; ^1^H NMR (400 MHz, CDCl_3_): δ ppm 0.99 (t, *J* = 7.28 Hz, 6H), 1.45–1.50 (m, 4H), 1.71 (s, 12H), 1.81–1.83 (m, 4H), 1.97–1.99 (m, 2H), 2.73–2.76 (m, 4H), 4.21 (t, *J* = 7.14 Hz, 4H), 6.25 (d, *J* = 14.1 Hz, 2H), 7.11 (d, *J* = 8.42 Hz, 2H), 7.31 (s, 2H), 7.35 (d, *J* = 8.42 Hz, 2H), 8.32 (d, *J* = 14.1 Hz, 2H); ^13^C NMR (100 MHz, CDCl_3_): δ ppm 13.9, 20.4, 20.7, 26.8, 28.1, 29.5, 45.2, 49.3, 102.0, 112.0, 122.8, 128.3, 128.9, 130.9, 141.0, 142.7, 144.2, 150.6, 171.8; λ_abs_ = 790 nm in MeOH; HRMS (ESI) Calcd. for [C_38_H_46_Cl_3_N_2_]^1+^*m*/*z* 637.1456, found *m*/*z* 637.1230.

#### 5-Chloro-2-((*E*)-2-((*E*)-2-chloro-3-(2-((*E*)-5-chloro-3,3-dimethyl-1-(3-phenylpropyl) indolin-2-ylidene)ethylidene)cyclohex-1-en-1-yl)vinyl)-3,3-dimethyl-1-(3-phenylpropyl)-3H-indol-1-ium Bromide **19**

Yield (49%, 0.15 g); mp 181–183 °C; ^1^H NMR (400 MHz, CDCl_3_): δ ppm 1.69 (s, 12H), 1.93 (br, 2H), 2.17–2.18 (m, 4H), 2.57 (br, 4H), 2.87–2.90 (m, 4H), 4.27 (t, *J* = 6.88 Hz, 4H), 6.26 (d, *J* = 14.1 Hz, 2H), 6.99 (d, J = 8.32 Hz, 2H), 7.33 (m, 14H), 8.25 (d, *J* = 14.1 Hz, 2H), ^13^C NMR (100 MHz, CDCl_3_): δ ppm 1.0, 15.3, 20.6, 28.0, 28.5, 32.7, 44.0, 49.3, 65.9, 101.9, 112.0, 122.7, 128.4, 128.6, 128.7, 129.0, 130.9, 140.3, 142.6, 144.1, 150.5, 171.5; λ_abs_ = 790 nm in MeOH.

#### 5-Bromo-2-((*E*)-2-((*E*)-3-(2-((*E*)-5-bromo-1-butyl-3,3-dimethylindolin-2-ylidene)ethylidene)-2-chlorocyclohex-1-en-1-yl)vinyl)-1-butyl-3,3-dimethyl-3H-indol-1-ium Iodide **20**

Yield (54%, 0.16 g); mp 152–154 °C; ^1^H NMR (400 MHz, DMSO-*d*_6_): δ ppm 0.95 (t, *J* = 7.34 Hz, 6H), 1.41–1.47 (m, 4H), 1.72 (s, 16H), 1.86–1.90 (m, 2H), 2.71–2.74 (m, 4H), 4.19 (t, *J* = 7.23 Hz, 4H), 6.31 (d, *J* = 14.1 Hz, 2H), 7.40 (d, *J* = 8.51 Hz, 2H), 7.60 (dd, *J* = 8.46 Hz, 1.71 Hz, 2H), 7.87 (d, *J* = 1.67 Hz, 2H), 8.27 (d, *J* = 14.1 Hz, 2H), ^13^C NMR (100 MHz, DMSO-*d*_6_): δ ppm 14.2, 19.9, 20.8, 26.3, 27.8, 29.6, 44.3, 49.6, 102.5, 113.9, 118.0, 126.3, 127.4, 131.9, 141.9, 143.6, 143.9, 148.9, 172.3; λ_abs_ = 795 nm in MeOH.

#### 1-Butyl-2-((*E*)-2-((*E*)-3-(2-((*E*)-1-butyl-3,3-dimethylindolin-2-ylidene)ethylidene)-2-chlorocyclohex-1-en-1-yl)vinyl)-3,3-dimethyl-3H-indol-1-ium Iodide **21**

Yield (72%, 0.22 g); mp > 200 °C; ^1^H NMR (400 MHz, CDCl_3_): δ ppm 1.01 (t, *J* = 7.26 Hz, 6H), 1.49–1.53 (m, 4H), 1.72 (s, 12H), 1.85–1.86 (m, 4H), 2.08 (br, 2H), 2.71–2.74 (m, 4H), 4.20 (t, *J* = 7.10 Hz, 4H), 6.21 (d, *J* = 14.1 Hz, 2H), 7.18 (d, *J* = 8.08 Hz, 2H), 7.26 (dd, *J* = 8.96 Hz, 7.44 Hz, 2H), 7.39 (d, *J* = 5.20 Hz, 4H), 8.35 (d, *J* = 14.1 Hz, 2H); ^13^C NMR (100 MHz, CDCl_3_): δ ppm 13.9, 20.4, 20.7, 26.7, 28.2, 29.5, 44.9, 49.4, 101.3, 111.0, 122.3, 125.4, 127.3, 128.8, 141.1, 142.4, 144.3, 150.5, 172.4; λ_abs_ = 780 nm in MeOH.

#### 1-Butyl-2-((1*E*,3*E*,5*E*)-7-((*E*)-1-butyl-5-chloro-3,3-dimethylindolin-2-ylidene)hepta-1,3,5-trien-1-yl)-5-chloro-3,3-dimethyl-3H-indol-1-ium Iodide **22**

Yield (51%, 0.14 g); mp 162–164 °C; ^1^H NMR (400 MHz, DMSO-*d*_6_): δ ppm 0.91 (t, *J* = 7.30 Hz, 6H), 1.35–1.40 (m, 4H), 1.63 (s, 16H), 4.05 (t, *J* = 7.03 Hz, 4H), 6.39 (d, *J* = 13.7 Hz, 2H), 6.57 (t, *J* = 12.6 Hz, 2H), 7.41 (dd, *J* = 10.9 Hz,1.80 Hz, 4H), 7.75 (br, 2H), 7.79–7.92 (m, 3H); ^13^C NMR (100 MHz, CDCl_3_): δ ppm 13.9, 20.3, 28.1, 29.5, 45.6, 49.2, 104.3, 111.3, 122.8, 127.1, 128.7, 130.3, 141.0, 142.7, 151.7, 154.2, 157.6, 171.0; λ_abs_ = 755 nm in MeOH; HRMS (ESI) calcd for [C_35_H_43_Cl_2_N_2_]^1+^*m*/*z* 562.6456, found *m*/*z* 561.2798.

#### 5-Chloro-2-((1E,3E,5E)-7-((E)-5-chloro-3,3-dimethyl-1-(3-phenylpropyl)indolin-2-ylidene) hepta-1,3,5-trien-1-yl)-3,3-dimethyl-1-(3-phenylpropyl)-3H-indol-1-ium Bromide **23**

Yield (48%, 0.13 g); mp 154–156 °C; ^1^H NMR (400 MHz, CDCl_3_): **δ** ppm 1.65 (br, 4H), 1.77 (br, 4H), 2.11–2.15 (m, 4H), 2.83–2.87 (m, 4H), 4.06–4.08 (m, 4H), 6.18 (d, *J* = 12.0 Hz, 1H), 6.32 (d, *J* = 13.6 Hz, 1H), 6.59 (t, *J* = 12.0 Hz, 1H), 6.77 (d, *J* = 13.5 Hz, 1H), 6.83 (d, *J* = 7.82 Hz, 1H), 7.27–7.25 (m, 12H), 7.28 (br, 4H), 7.64–7.62 (m,1H), 7.81–7.75 (m, 1H), 8.35 (t, *J* = 13.0 Hz, 1H); ^13^C NMR (100 MHz, CDCl_3_): **δ** ppm 27.9, 28.0, 28.6, 32.7, 43.7, 49.0, 49.5, 104.3, 104.6, 111.3, 122.8, 122.9, 128.7, 140.3, 140.6, 140.8, 142.6, 143.1, 154.8, 172.8; λ_abs_ = 755 nm in MeOH.

#### 5-Bromo-2-((1E,3E,5E)-7-((E)-5-bromo-3,3-dimethyl-1-(3-phenylpropyl)indolin-2-ylidene)hepta-1,3,5-trien-1-yl)-3,3-dimethyl-1-(3-phenylpropyl)-3H-indol-1-ium Bromide **24**

Yield (41%, 0.11 g); mp 158–160 °C; ^1^H NMR (400 MHz, CDCl_3_): **δ** ppm 1.64 (s, 12H), 2.09–2.13 (m, 4H), 2.84–2.86 (m, 4H), 4.06–4.08 (m, 4H), 6.18 (d, *J* = 12.8 Hz, 1H), 6.30 (d, *J* = 13.6 Hz, 1H), 6.59 (t, *J* = 12.0 Hz, 1H), 6.79 (d, *J* = 7.77 Hz, 2H), 7.26–7.39 (m, 16H), 7.62–7.65 (m, 1H), 7.74–7.80 (m, 1H), 8.37 (t, *J* = 13.0, 1H); ^13^C NMR (100 MHz, CDCl_3_): **δ** ppm 28.0, 32.7, 43.6, 49.0, 49.5, 104.3, 111.7, 117.8, 118.1, 125.6, 125.7, 126.5, 128.6, 128.7, 131.4, 131.6, 140.3, 141.1, 141.3, 142.9, 143.4, 151.5, 154.8, 170.5, 172.6; λ_abs_ = 755 nm in MeOH.

#### 5-Bromo-2-((1E,3E,5E)-7-((E)-5-bromo-1-butyl-3,3-dimethylindolin-2-ylidene)hepta-1,3,5-trien-1-yl)-1-butyl-3,3-dimethyl-3H-indol-1-ium Iodide **25**

Yield (45%, 0.13 g); mp 160–162 °C; ^1^H NMR (400 MHz, CDCl_3_): δ ppm 0.95 (t, *J* = 7.30 Hz, 6H), 1.41–1.47 (m, 4H), 1.66 (s, 12H), 1.72–1.76 (m, 4H), 4.00 (br, 4H), 6.20 (d, *J* = 13.2 Hz, 2H), 6.64 (t, *J* = 12.3 Hz, 2H), 6.99 (d, *J* = 8.35 Hz, 2H), 7.40–7.45 (m, 4H), 7.80–7.83 (m, 3H); ^13^C NMR (100 MHz, CDCl_3_): δ ppm 13.9, 20.3, 28.1, 29.4, 44.6, 49.1, 104.2, 112.0, 117.8, 118.1, 125.6, 127.0, 131.6, 141.4, 143.0, 151.7, 157.6, 170.8; λ_abs_ = 755 nm in MeOH.

#### 5-Chloro-2-((1E,3Z)-3-chloro-5-((E)-5-chloro-3,3-dimethyl-1-(3-phenylpropyl)indolin-2-ylidene)penta-1,3-dien-1-yl)-3,3-dimethyl-1-(3-phenylpropyl)-3H-indol-1-ium Bromide **26**

Yield (63%, 0.18 g); mp 161–163 °C; ^1^H NMR (400 MHz, CDCl_3_): δ ppm 1.98 (s, 12H), 2.18–2.21 (m, 4H), 2.83–2.86 (m, 4H), 4.02 (t, *J* = 15.3 Hz, 4H), 6.23 (d, *J* = 13.4 Hz, 2H), 6.81 (d, *J* = 8.43 Hz, 2H), 7.27–7.36 (m, 12H), 7.38–7.40 (m, 4H), 9.32 (d, *J* = 13.4 Hz, 2H); ^13^C NMR (100 MHz, CDCl_3_): δ ppm 27.6, 28.2, 33.0, 43.9, 50.4, 103.0, 111.6, 118.9, 119.4, 126.2, 126.7, 128.4, 128.9, 131.2, 139.7, 140.7, 144.5, 152.2, 175.3; λ_abs_ = 656 nm in MeOH.

#### 1-Butyl-2-((1E,3Z)-5-((E)-1-butyl-5-chloro-3,3-dimethylindolin-2-ylidene)-3-chloropenta-1,3-dien-1-yl)-5-chloro-3,3-dimethyl-3H-indol-1-ium Iodide **27**

Yield (71%, 0.20 g); mp > 200 °C; ^1^H NMR (400 MHz, CDCl_3_): **δ** ppm 1.03–0.99 (t, *J* = 13.8 Hz, 6H), 1.51–1.47 (m, 4H), 1.85–1.82 (m, 4H), 1.92 (s, 12H), 4.12–4.10 (m, 4H), 6.39 (d, *J* = 13.3 Hz, 2H), 7.16 (d, *J* = 8.02 Hz, 2H), 7.33–7.35 (m, 4H), 8.76 (d, *J* = 13.3 Hz, 2H); ^13^C NMR (100 MHz, CDCl_3_): **δ** ppm 13.8, 20.3, 27.9, 29.3, 44.7, 50.3, 100.9, 112.1, 123.1, 125.0, 128.7, 131.4, 140.3, 143.8, 148.7, 174.6; λ_abs_ = 660 nm in MeOH.

#### 5-Bromo-2-((1E,3Z)-3-bromo-5-((E)-5-bromo-3,3-dimethyl-1-(3-phenylpropyl)indolin-2-ylidene)penta-1,3-dien-1-yl)-3,3-dimethyl-1-(3-phenylpropyl)-3H-indol-1-ium Bromide **28**

Yield (51%, 0.15 g); mp 163–165 °C; ^1^H NMR (400 MHz, CDCl_3_): δ ppm 1.95 (s, 12H), 2.2–2.1 (m, 4H), 2.85–2.82 (m, 4H), 4.01 (t, *J* = 14.4 Hz, 4H), 6.26 (d, *J* = 13.2 Hz, 2H), 6.78 (d, *J* = 8.32 Hz, 2H), 7.30–7.24 (m, 8H), 7.38–7.35 (m, 4H), 7.46–7.42 (m, 4H), 9.37 (d, *J* = 13.2 Hz, 2H); ^13^C NMR (100 MHz, CDCl_3_): δ ppm 27.6, 28.2, 33.0, 43.9, 50.4, 103.0, 111.6, 118.9, 119.4, 126.2, 126.7, 128.4, 128.9, 131.2, 139.7, 140.7, 144.5, 153.3, 175.3; λ_abs_ = 650 nm in MeOH.

## Supporting Information Available

Additional figures and table illustrating absorbance profiles of fluorophores **18–28** at various concentrations in six different solvents, molar extinction coefficient of fluorophores **18–28** in DMSO, % fluorescence intensity change with time of fluorophores **18–20, 24, 25**, and **28**, and ^1^H and ^13^C NMR of **18–28** (PDF)
Molecular formula strings of the reported compounds (CSV)

## Abbrevations

MRI: magnetic resonance imaging
US: ultrasound
CT: computed tomography
PET: positron emission tomography
SPECT: single-photon emission computed tomography
NIR: near-infrared
DCM: dichloromethane
NMR: nuclear magnetic resonance
DMSO: dimethyl sulfoxide
MeOH: methanol
EtOH: ethanol
TBR: tumor-to-background ratio
EtOAc: ethyl acetate
AcONa: sodium acetate
TPSA: total polar surface area
nrotb: number of rotatable bands
FMO: frontier molecular orbital
DFT: density functional theory
MeCN: acetonitrile
HOMO: highest occupied molecular orbital
LUMO: lowest unoccupied molecular orbital
SBR: signal-to-background ratio

## References

[ref1] Weissleder R.; Pittet M. J. Imaging in the era of molecular oncology. Nature 2008, 452, 580–589. 10.1038/nature06917. 18385732 PMC2708079

[ref2] Frangioni J. V. New technologies for human cancer imaging. J. Clin. Oncol. 2008, 26, 4012–4021. 10.1200/jco.2007.14.3065. 18711192 PMC2654310

[ref3] aMislow J. M.; Golby A. J.; Black P. M. Origins of intraoperative MRI. Neurosurg. Clin. North Am. 2009, 20, 137–146. 10.1016/j.nec.2009.04.002. PMC2902263 19555875

[ref4] Gioux S.; Choi H. S.; Frangioni J. V. Image-guided surgery using invisible near-infrared light: fundamentals of clinical translation. Mol. Imaging 2010, 9, 237–255. 10.2310/7290.2010.00034. 20868625 PMC3105445

[ref5] Chan H. H. L.; Siewerdsen J. H.; Vescan A.; Daly M. J.; Prisman E.; Irish J. C. 3D Rapid Prototyping for Otolaryngology-Head and Neck Surgery: Applications in Image-Guidance, Surgical Simulation and Patient-Specific Modeling. PLoS One 2015, 10, e0136370 10.1371/journal.pone.0136370. 26331717 PMC4557980

[ref6] Schaafsma B. E.; Mieog J. S.; Hutteman M.; van der Vorst J. R.; Kuppen P. J.; Löwik C. W.; Frangioni J. V.; van de Velde C. J.; Vahrmeijer A. L. The clinical use of indocyanine green as a near-infrared fluorescent contrast agent for image-guided oncologic surgery. J. Surg. Oncol. 2011, 104, 323–332. 10.1002/jso.21943. 21495033 PMC3144993

[ref7] Hong G.; Antaris A. L.; Dai H. Near-infrared fluorophores for biomedical imaging. Nat. Biomed. Eng. 2017, 1, 0010 10.1038/s41551-016-0010.

[ref8] aAntaris A. L.; Chen H.; Cheng K.; Sun Y.; Hong G.; Qu C.; Diao S.; Deng Z.; Hu X.; Zhang B.; et al. A small-molecule dye for NIR-II imaging. Nat. Mater. 2016, 15, 235–242. 10.1038/nmat4476. 26595119

[ref9] avan der Vorst J. R.; Vahrmeijer A. L.; Hutteman M.; Bosse T.; Smit V. T.; van de Velde C. J.; Frangioni J. V.; Bonsing B. A. Near-infrared fluorescence imaging of a solitary fibrous tumor of the pancreas using methylene blue. World J. Gastrointest. Surg. 2012, 4, 180–184. 10.4240/wjgs.v4.i7.180. 22905287 PMC3420986

[ref10] Soriano E.; Outler L.; Owens E. A.; Henary M. Synthesis of Asymmetric Monomethine Cyanine Dyes with Red-Shifted Optical Properties. J. Heterocycl. Chem. 2015, 52, 180–184. 10.1002/jhet.1963.

[ref11] Levitz A.; Marmarchi F.; Henary M. Introduction of various substitutions to the methine bridge of heptamethine cyanine dyes Via substituted dianil linkers. Photochem. Photobiol. Sci. 2018, 17, 1409–1416. 10.1039/c8pp00218e. 30234861 PMC6193477

[ref12] aBandi V. G.; Luciano M. P.; Saccomano M.; Patel N. L.; Bischof T. S.; Lingg J. G. P.; Tsrunchev P. T.; Nix M. N.; Ruehle B.; Sanders C.; et al. Targeted multicolor in vivo imaging over 1,000 nm enabled by nonamethine cyanines. Nat. Methods 2022, 19, 353–358. 10.1038/s41592-022-01394-6. 35228725

[ref13] Owens E. A.; Hyun H.; Tawney J. G.; Choi H. S.; Henary M. Correlating Molecular Character of NIR Imaging Agents with Tissue-Specific Uptake. J. Med. Chem. 2015, 58, 4348–4356. 10.1021/acs.jmedchem.5b00475. 25923454 PMC4603651

[ref14] Lapins M.; Arvidsson S.; Lampa S.; Berg A.; Schaal W.; Alvarsson J.; Spjuth O. A confidence predictor for log D using conformal regression and a support-vector machine. J. Cheminf. 2018, 10, 17 10.1186/s13321-018-0271-1. PMC5882484 29616425

[ref15] Waring M. J. Lipophilicity in drug discovery. Expert Opin. Drug Discovery 2010, 5, 235–248. 10.1517/17460441003605098. 22823020

[ref16] aWaring M. J. Defining optimum lipophilicity and molecular weight ranges for drug candidates-Molecular weight dependent lower log D limits based on permeability. Bioorg. Med. Chem. Lett. 2009, 19, 2844–2851. 10.1016/j.bmcl.2009.03.109. 19361989

[ref17] Álamo P.; Pallarès V.; Céspedes M. V.; Falgàs A.; Sanchez J. M.; Serna N.; Sánchez-García L.; Voltà-Duràn E.; Morris G. A.; Sánchez-Chardi A.; et al. Fluorescent Dye Labeling Changes the Biodistribution of Tumor-Targeted Nanoparticles. Pharmaceutics 2020, 12, 1004 10.3390/pharmaceutics12111004. 33105866 PMC7690626

[ref18] aChoi H. S.; Frangioni J. V. Nanoparticles for biomedical imaging: fundamentals of clinical translation. Mol. Imaging 2010, 9, 291–310. 10.2310/7290.2010.00031. 21084027 PMC3017480

[ref19] Owens E. A.; Henary M.; El Fakhri G.; Choi H. S. Tissue-Specific Near-Infrared Fluorescence Imaging. Acc. Chem. Res. 2016, 49, 1731–1740. 10.1021/acs.accounts.6b00239. 27564418 PMC5776714

[ref20] Owens E. A.; Lee S.; Choi J.; Henary M.; Choi H. S. NIR fluorescent small molecules for intraoperative imaging. Wiley Interdiscip. Rev.: Nanomed. Nanobiotechnol. 2015, 7, 828–838. 10.1002/wnan.1337. 25645081 PMC4520803

[ref21] aHyun H.; Owens E. A.; Wada H.; Levitz A.; Park G.; Park M. H.; Frangioni J. V.; Henary M.; Choi H. S. Cartilage-Specific Near-Infrared Fluorophores for Biomedical Imaging. Angew. Chem., Int. Ed. 2015, 54, 8648–8652. 10.1002/anie.201502287. PMC4504790 26095685

[ref22] Kang H.; Shamim M.; Yin X.; Adluru E.; Fukuda T.; Yokomizo S.; Chang H.; Park S. H.; Cui Y.; Moy A. J.; et al. Tumor-Associated Immune-Cell-Mediated Tumor-Targeting Mechanism with NIR-II Fluorescence Imaging. Adv. Mater. 2022, 34, 2106500 10.1002/adma.202106500. PMC8881361 34913533

[ref23] Flanagan J. H. Jr.; Khan S. H.; Menchen S.; Soper S. A.; Hammer R. P. Functionalized tricarbocyanine dyes as near-infrared fluorescent probes for biomolecules. Bioconjugate Chem. 1997, 8, 751–756. 10.1021/bc970113g. 9327141

[ref24] Owens E. A.; Hyun H.; Dost T. L.; Lee J. H.; Park G.; Pham D. H.; Park M. H.; Choi H. S.; Henary M. Near-Infrared Illumination of Native Tissues for Image-Guided Surgery. J. Med. Chem. 2016, 59, 5311–5323. 10.1021/acs.jmedchem.6b00038. 27100476 PMC5733074

[ref25] Ertl P.; Rohde B.; Selzer P. Fast Calculation of Molecular Polar Surface Area as a Sum of Fragment-Based Contributions and Its Application to the Prediction of Drug Transport Properties. J. Med. Chem. 2000, 43, 3714–3717. 10.1021/jm000942e. 11020286

[ref26] aPrasanna S.; Doerksen R. J. Topological polar surface area: a useful descriptor in 2D-QSAR. Curr. Med. Chem. 2009, 16, 21–41. 10.2174/092986709787002817. 19149561 PMC7549127

[ref27] Buabeng E. R.; Dinh J.; Fukuda T.; Kang H.; Kashiwagi S.; Choi H. S.; Henary M. Microwave-Assisted Synthesis of the Red-Shifted Pentamethine Tetrahydroxanthylium Core with Absorbance within the Near Infrared-II Window. ACS Pharmacol. Transl. Sci. 2022, 5, 963–972. 10.1021/acsptsci.2c00121. 36268114 PMC9578133

[ref28] Mohammad-Jafarieh P.; Akbarzadeh A.; Salamat-Ahangari R.; Pourhassan-Moghaddam M.; Jamshidi-Ghaleh K. Solvent effect on the absorption and emission spectra of carbon dots: evaluation of ground and excited state dipole moment. BMC Chem. 2021, 15, 53 10.1186/s13065-021-00779-6. 34563252 PMC8587513

[ref29] Henary M.; Mojzych M. Stability and Reactivity of Polymethine Dyes in Solution. In Heterocyclic Polymethine Dyes: Synthesis, Properties and Applications; Strekowski L., Ed.; Springer: Berlin Heidelberg, 2008; pp 221–238.

[ref30] Kang J.; Kaczmarek O.; Liebscher J.; Dähne L. Prevention of H-Aggregates Formation in Cy5 Labeled Macromolecules. Int. J. Polym. Sci. 2010, 2010, 264781 10.1155/2010/264781.

[ref31] Mapp C. T.; Owens E. A.; Henary M.; Grant K. B. Oxidative cleavage of DNA by pentamethine carbocyanine dyes irradiated with long-wavelength visible light. Bioorg. Med. Chem. Lett. 2014, 24, 214–219. 10.1016/j.bmcl.2013.11.035. 24332091

[ref32] St Lorenz A.; Buabeng E. R.; Taratula O.; Taratula O.; Henary M. Near-Infrared Heptamethine Cyanine Dyes for Nanoparticle-Based Photoacoustic Imaging and Photothermal Therapy. J. Med. Chem. 2021, 64, 8798–8805. 10.1021/acs.jmedchem.1c00771. 34081463 PMC10807376

[ref33] Laramie M. D.; Fouts B. L.; MacCuaig W. M.; Buabeng E.; Jones M. A.; Mukherjee P.; Behkam B.; McNally L. R.; Henary M. Improved pentamethine cyanine nanosensors for optoacoustic imaging of pancreatic cancer. Sci. Rep. 2021, 11, 4366 10.1038/s41598-021-83658-3. 33623069 PMC7902650

[ref34] Xiao Q.; Chen T.; Chen S. Fluorescent contrast agents for tumor surgery. Exp. Ther. Med. 2018, 16, 1577–1585. 10.3892/etm.2018.6401. 30186374 PMC6122374

[ref35] Buabeng E. R.; Henary M. 2-((E)-2-((E)-4-Chloro-5-(2-((E)-5-methoxy-3,3-dimethyl-1-(3-phenylpropyl)indolin-2-ylidene)ethylidene)-1,1-dimethyl-1,2,5,6-tetrahydropyridin-1-ium-3-yl)vinyl)-5-methoxy-3,3-dimethyl-1-(3-phenylpropyl)-3H-indol-1-ium. Molbank 2021, 2021, M1270 10.3390/M1270.

[ref36] Li D. H.; Smith B. D. Deuterated Indocyanine Green (ICG) with Extended Aqueous Storage Shelf-Life: Chemical and Clinical Implications. Chem. – Eur. J. 2021, 27, 14535–14542. 10.1002/chem.202102816. 34403531 PMC8530945

[ref37] Hyun H.; Park M. H.; Owens E. A.; Wada H.; Henary M.; Handgraaf H. J.; Vahrmeijer A. L.; Frangioni J. V.; Choi H. S. Structure-inherent targeting of near-infrared fluorophores for parathyroid and thyroid gland imaging. Nat. Med. 2015, 21, 192–197. 10.1038/nm.3728. 25559343 PMC4319985

