# Supplementary material for: Synthesis, Optical Properties, and In Vivo Biodistribution Performance of Polymethine Cyanine Fluorophores
Source: ACS Pharmacol Transl Sci. 2023 Jul 25;6(8):1192–206. doi: 10.1021/acsptsci.3c00101 (PMC10425993; doi:10.1021/acsptsci.3c00101)
Supplement: Supplementary file 1 — pt3c00101_si_001.pdf [file pt3c00101_si_001.pdf]

Supplementary Information (SI)

Synthesis, Optical Properties, and In Vivo Biodistribution Performance of Polymethine Cyanine Fluorophores

Md Shamim,[1] Jason Dinh[3], Chengeng Yang[3], Shinsuke Nomura[3], Satoshi Kashiwagi[3], Homan Kang[3], Hak Soo Choi[3]*, Maged Henary[1,2,] *

[1] Department of Chemistry, [2]Center of Diagnostics and Therapeutics, Georgia State University, Atlanta, GA 30303

[3]Gordon Center for Medical Imaging, Department of Radiology, Massachusetts General Hospital and Harvard Medical School, Boston, MA 02114

*Corresponding authors: Maged Henary, Email: mhenary1@gsu.edu and Hak Soo Choi, Email: hchoi12@mgh.harvard.edu

Contents of SI

Figure S1-S11 absorbance profiles of fluorophores **18-28** in two organic solvents (EtOH, DMSO) and two buffer solutions (HEPES, PBS)

Figure S12 molar extinction coefficients of fluorophores **18-28** in DMSO

Table S13 Percentage fluorescence intensities change with time

Figure S14-S35 NMR spectra

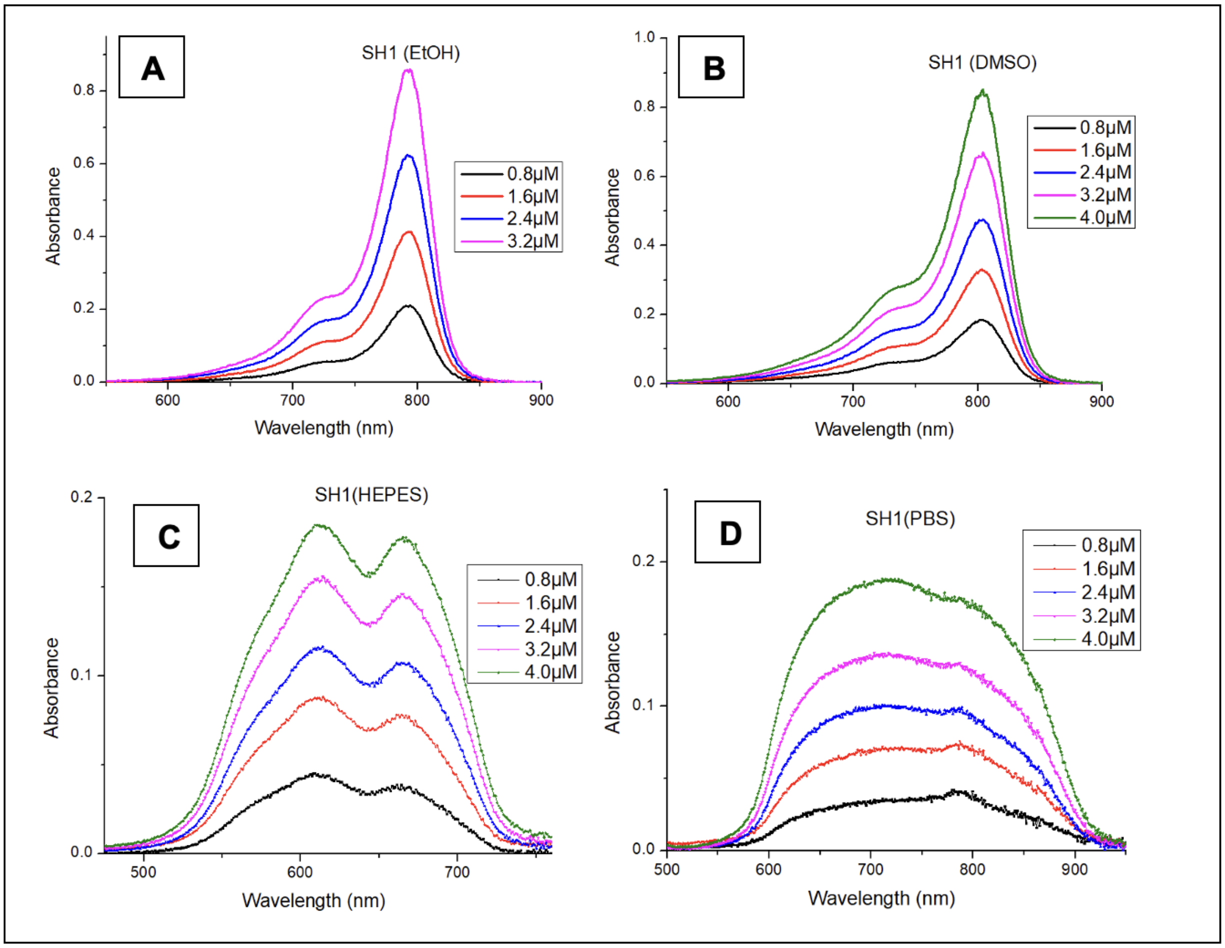

Figure S1. Concentrations dependent absorbances of fluorophore **18** in two organic solvents (EtOH, DMSO) and two buffer solutions (HEPES, PBS). The absorbance profiles of the **18** were recorded at various concentrations (0.8 µM, 1.6 µM, 2.4 µM, 3.2 µM and 4.0 µM) and the absorbance values obtained were plotted against wavelengths. Four media were used (A) EtOH, (B) DMSO, (C) HEPES, and (D) PBS.

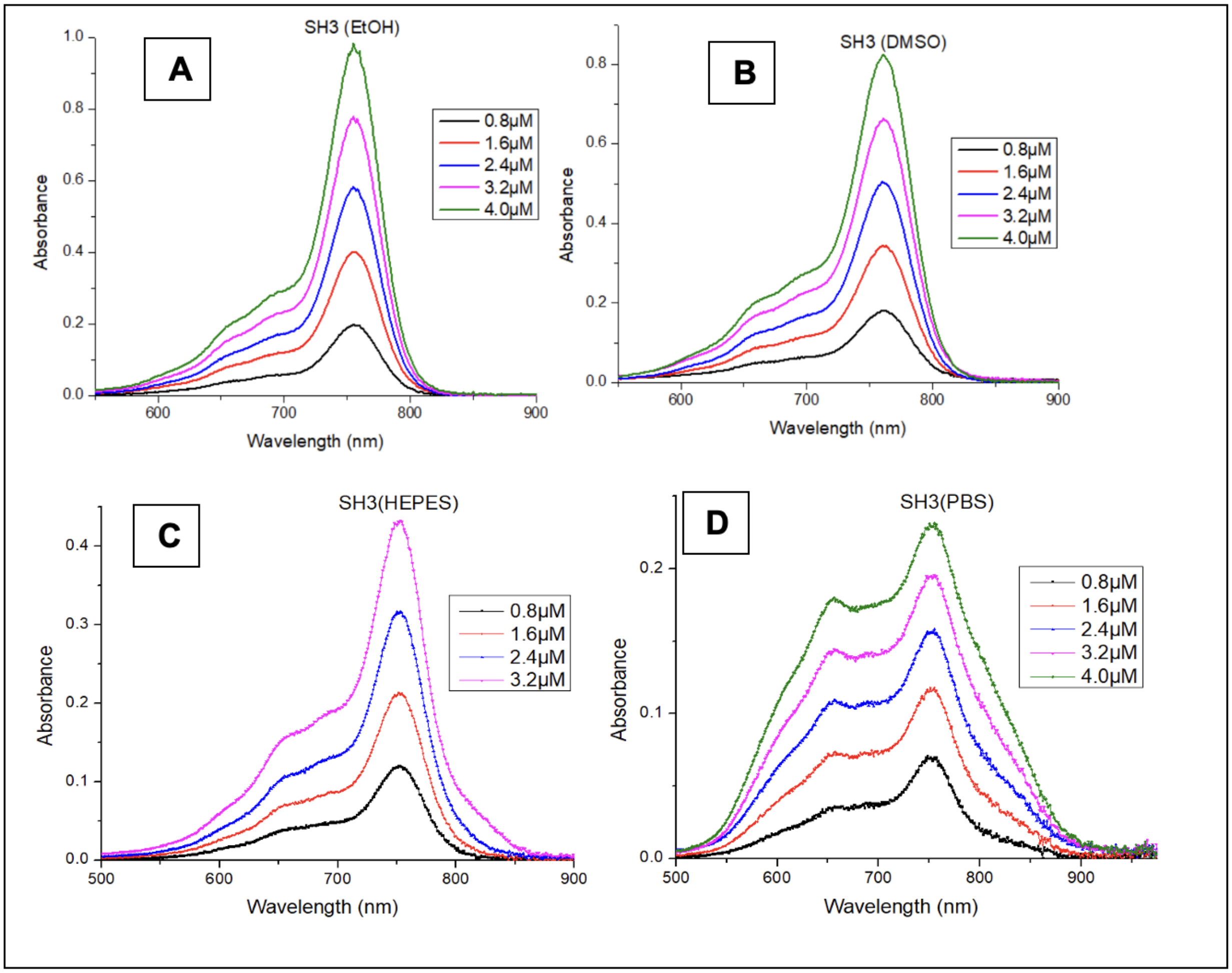

Figure S2. Concentrations dependent absorbances of fluorophore **19** in two organic solvents (EtOH, DMSO) and two buffer solutions (HEPES, PBS). The absorbance profiles of the **19** were recorded at various concentrations (0.8 μM, 1.6 μM, 2.4 μM, 3.2 μM and 4.0 μM) and the absorbance values obtained were plotted against wavelengths. Four media were used (A) EtOH, (B) DMSO, (C) HEPES, and (D) PBS.

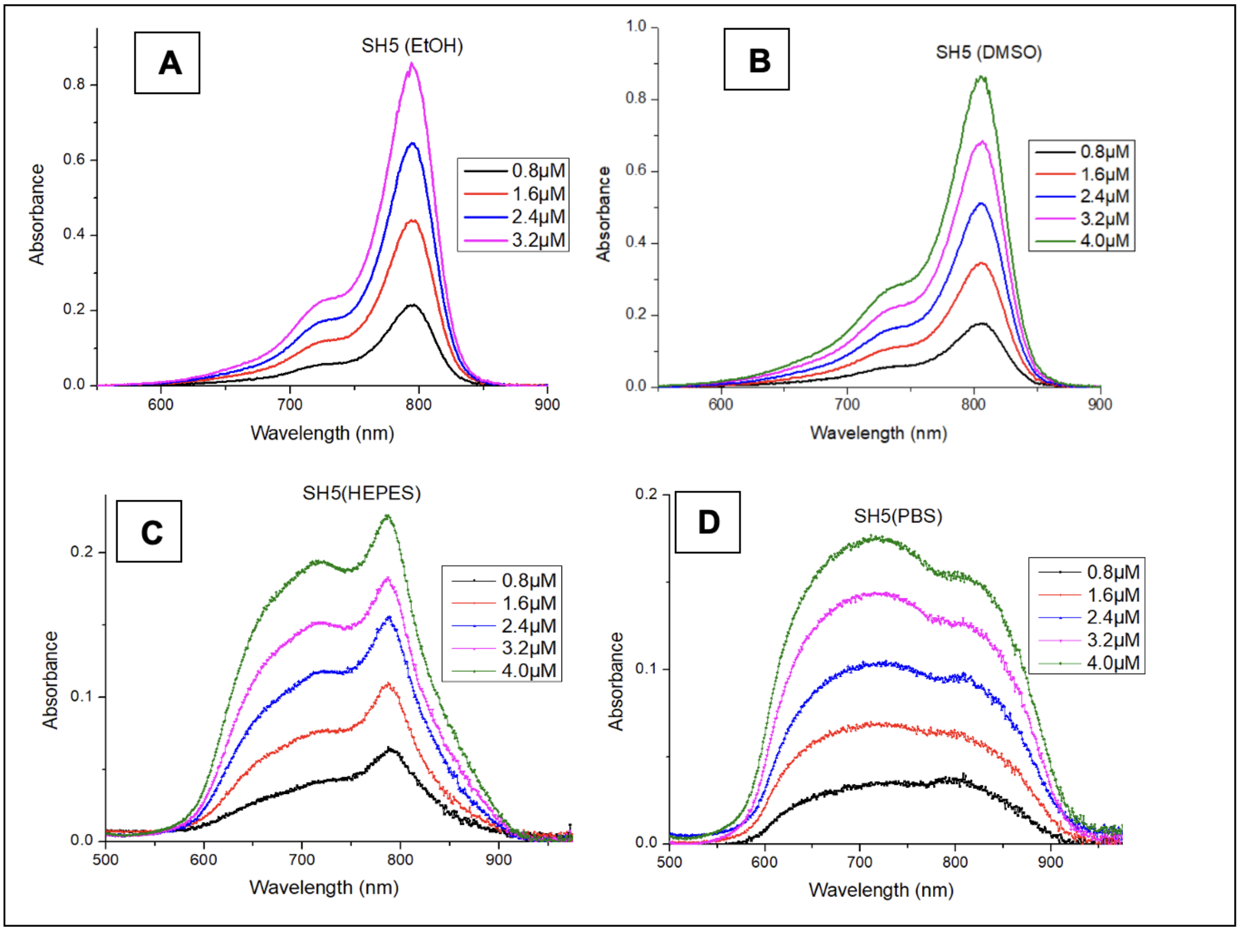

Figure S3. Concentrations dependent absorbances of fluorophore **20** in two organic solvents (EtOH, DMSO) and two buffer solutions (HEPES, PBS). The absorbance profiles of the **20** were recorded at various concentrations (0.8 µM, 1.6 µM, 2.4 µM, 3.2 µM and 4.0 µM) and the absorbance values obtained were plotted against wavelengths. Four media were used (A) EtOH, (B) DMSO, (C) HEPES, and (D) PBS.

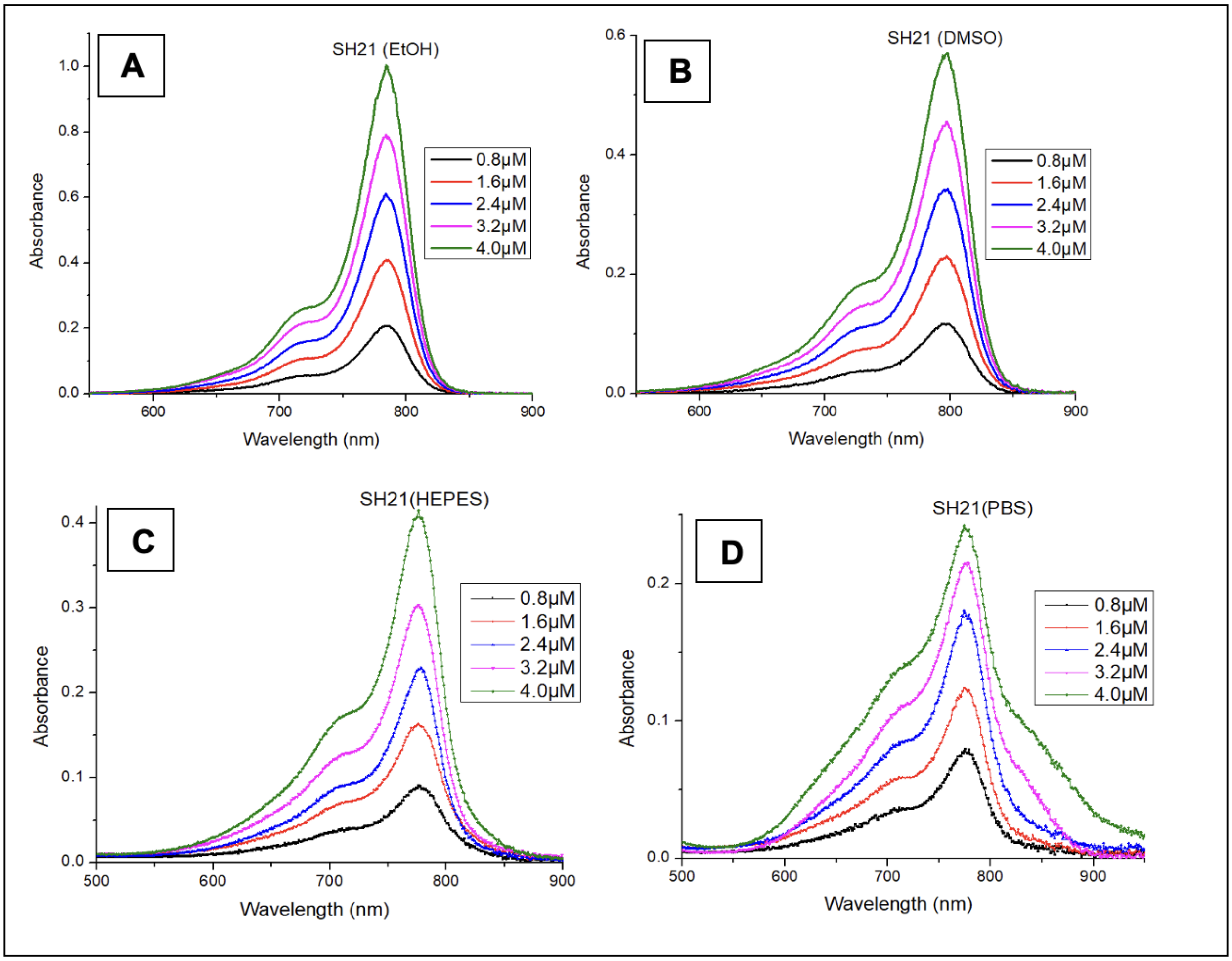

Figure S4. Concentrations dependent absorbances of fluorophore **21** in two organic solvents (EtOH, DMSO) and two buffer solutions (HEPES, PBS). The absorbance profiles of the **21** were recorded at various concentrations (0.8 µM, 1.6 µM, 2.4 µM, 3.2 µM and 4.0 µM) and the absorbance values obtained were plotted against wavelengths. Four media were used (A) EtOH, (B) DMSO, (C) HEPES, and (D) PBS.

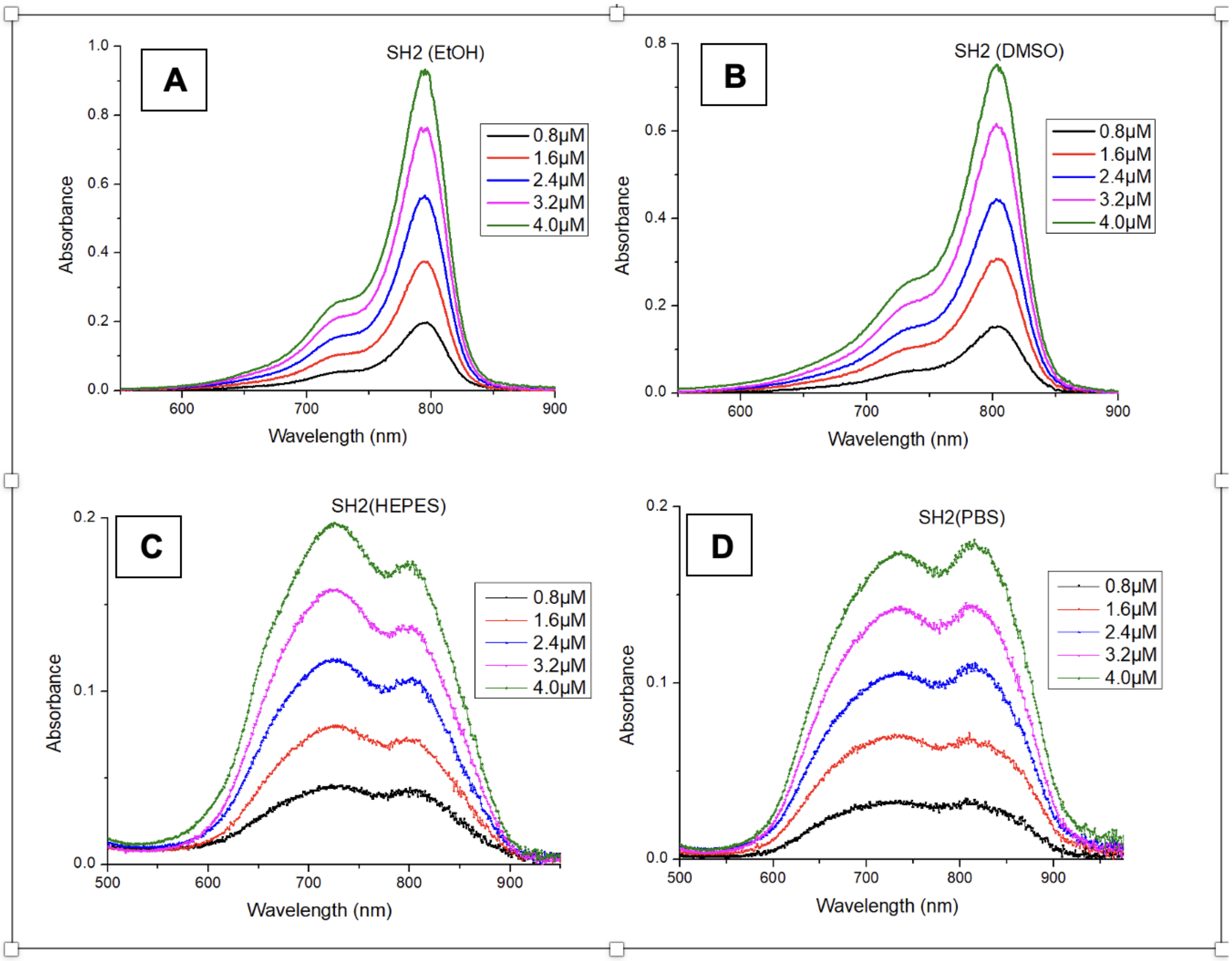

Figure S5. Concentrations dependent absorbances of fluorophore **22** in two organic solvents (EtOH, DMSO) and two buffer solutions (HEPES, PBS). The absorbance profiles of the **22** were recorded at various concentrations (0.8 μM, 1.6 μM, 2.4 μM, 3.2 μM and 4.0 μM) and the absorbance values obtained were plotted against wavelengths. Four media were used (A) EtOH, (B) DMSO, (C) HEPES, and (D) PBS.

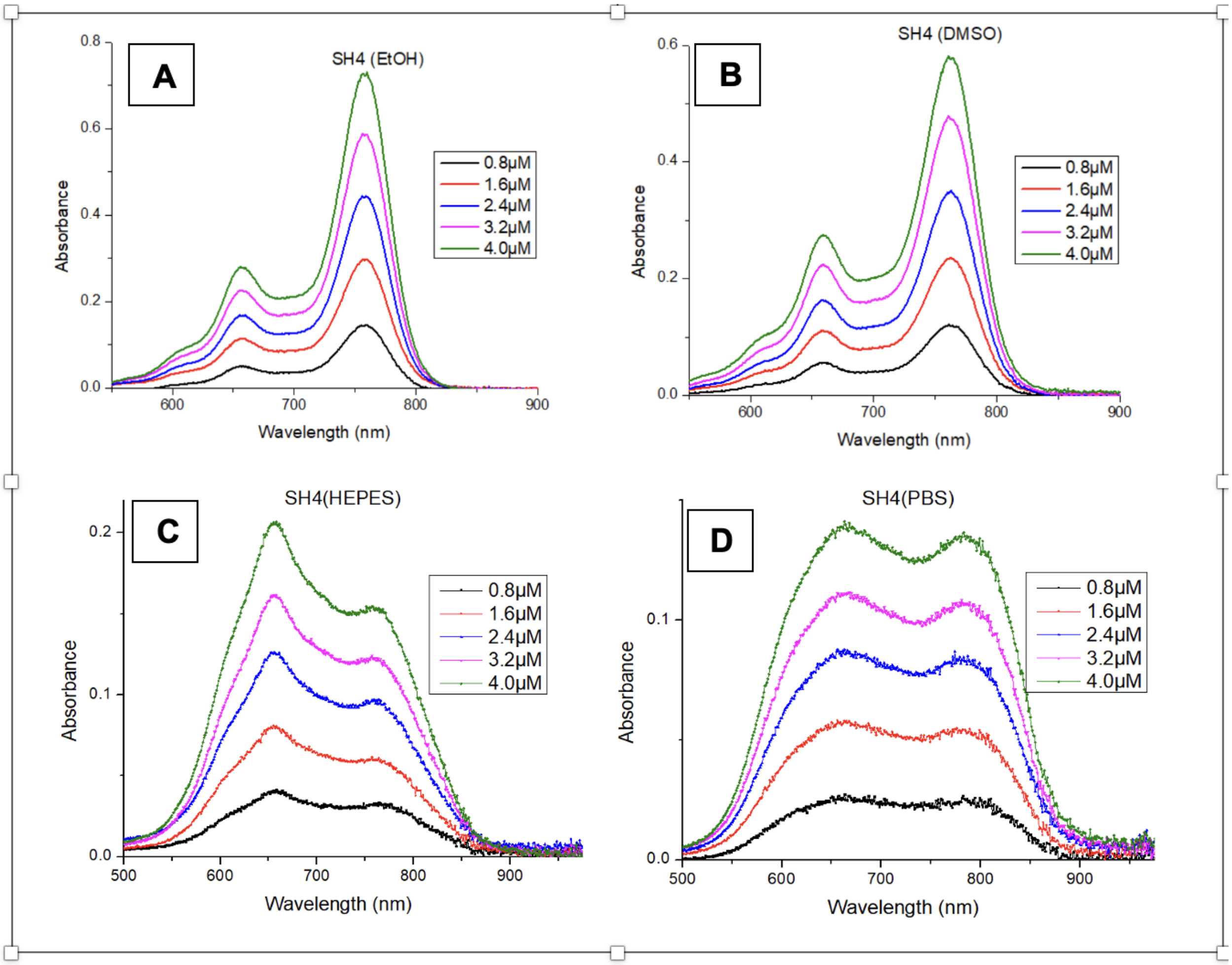

Figure S6. Concentrations dependent absorbances of fluorophore **23** in two organic solvents (EtOH, DMSO) and two buffer solutions (HEPES, PBS). The absorbance profiles of the **23** were recorded at various concentrations (0.8 µM, 1.6 µM, 2.4 µM, 3.2 µM and 4.0 µM) and the absorbance values obtained were plotted against wavelengths. Four media were used (A) EtOH, (B) DMSO, (C) HEPES, and (D) PBS.

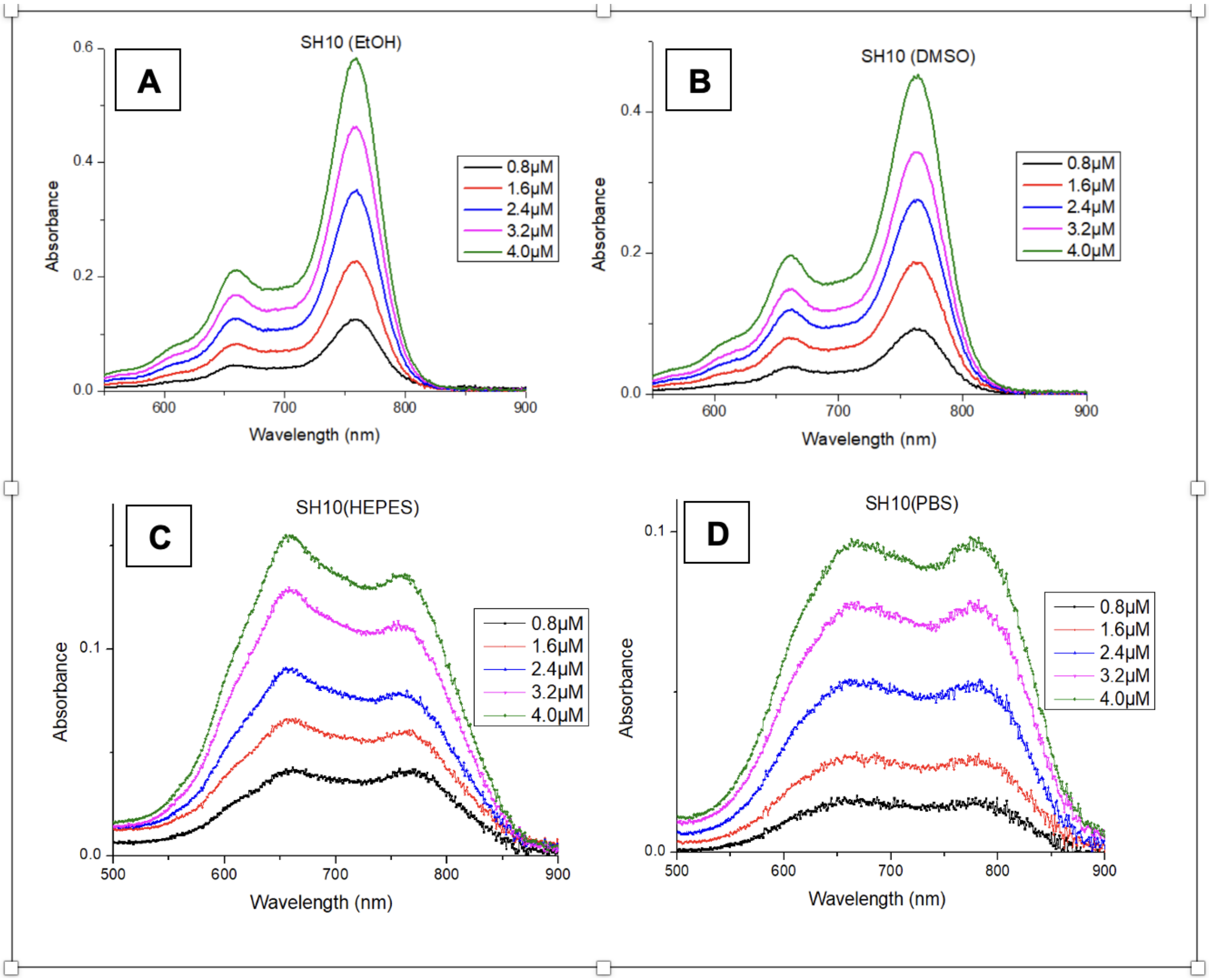

Figure S7. Concentrations dependent absorbances of fluorophore **24** in two organic solvents (EtOH, DMSO) and two buffer solutions (HEPES, PBS). The absorbance profiles of the **24** were recorded at various concentrations (0.8 µM, 1.6 µM, 2.4 µM, 3.2 µM and 4.0 µM) and the absorbance values obtained were plotted against wavelengths. Four media were used (A) EtOH, (B) DMSO, (C) HEPES, and (D) PBS.

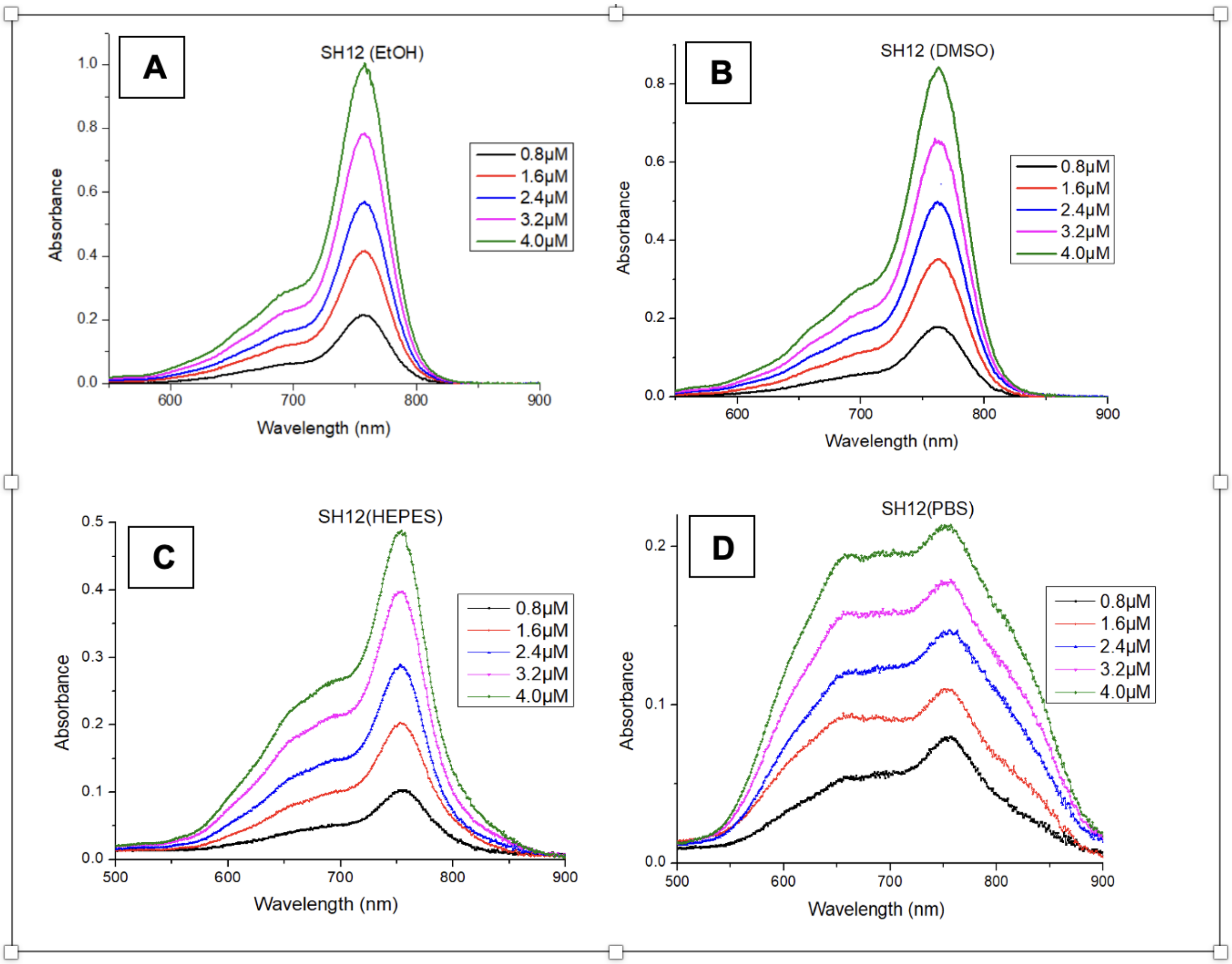

Figure S8. Concentrations dependent absorbances of fluorophore **25** in two organic solvents (EtOH, DMSO) and two buffer solutions (HEPES, PBS). The absorbance profiles of the **25** were recorded at various concentrations (0.8 µM, 1.6 µM, 2.4 µM, 3.2 µM and 4.0 µM) and the absorbance values obtained were plotted against wavelengths. Four media were used (A) EtOH, (B) DMSO, (C) HEPES, and (D) PBS.

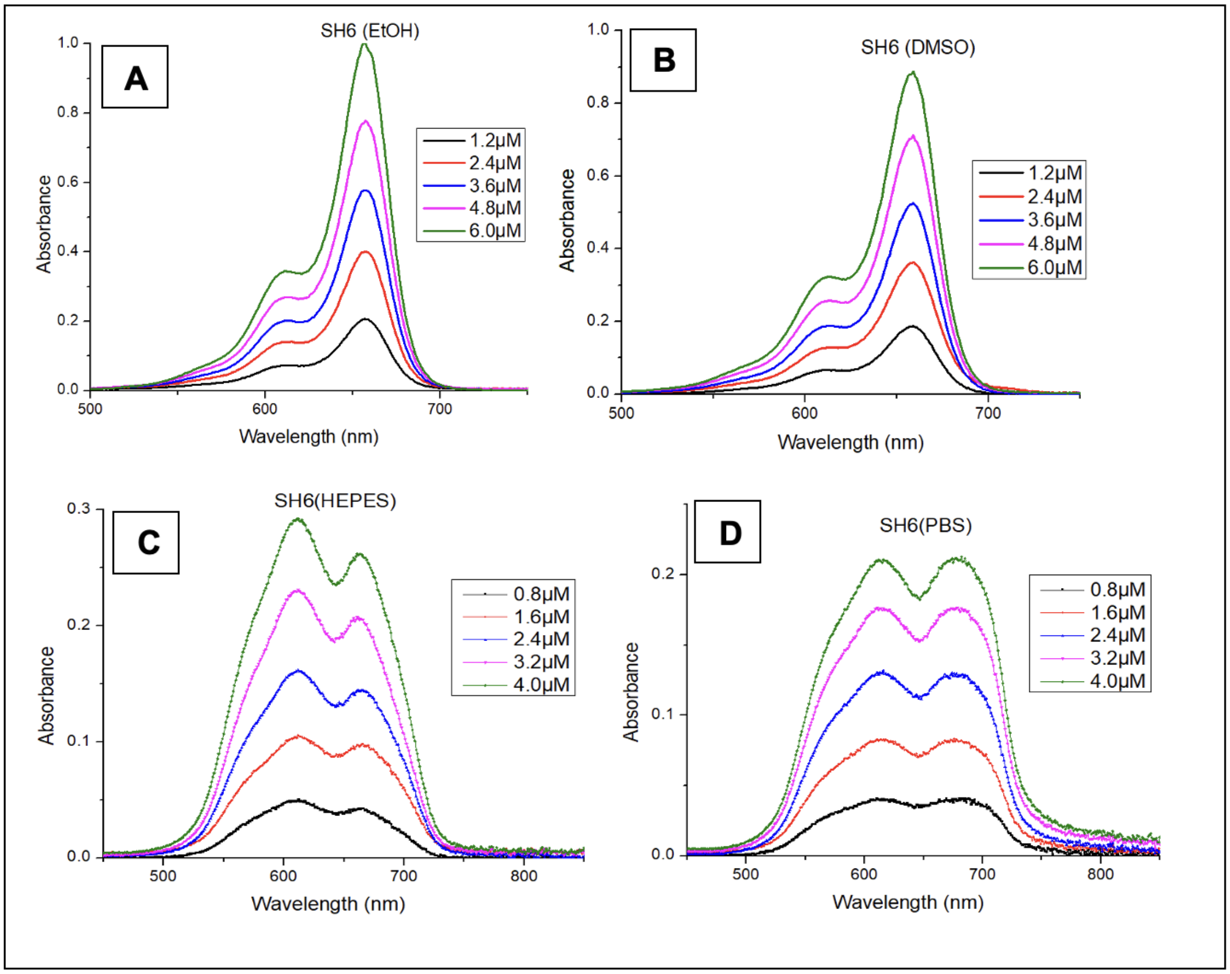

Figure S9. Concentrations dependent absorbances of fluorophore **26** in two organic solvents (EtOH, DMSO) and two buffer solutions (HEPES, PBS). The absorbance profiles of the **26** were recorded at various concentrations (0.8 µM, 1.6 µM, 2.4 µM, 3.2 µM and 4.0 µM) and the absorbance values obtained were plotted against wavelengths. Four media were used (A) EtOH, (B) DMSO, (C) HEPES, and (D) PBS.

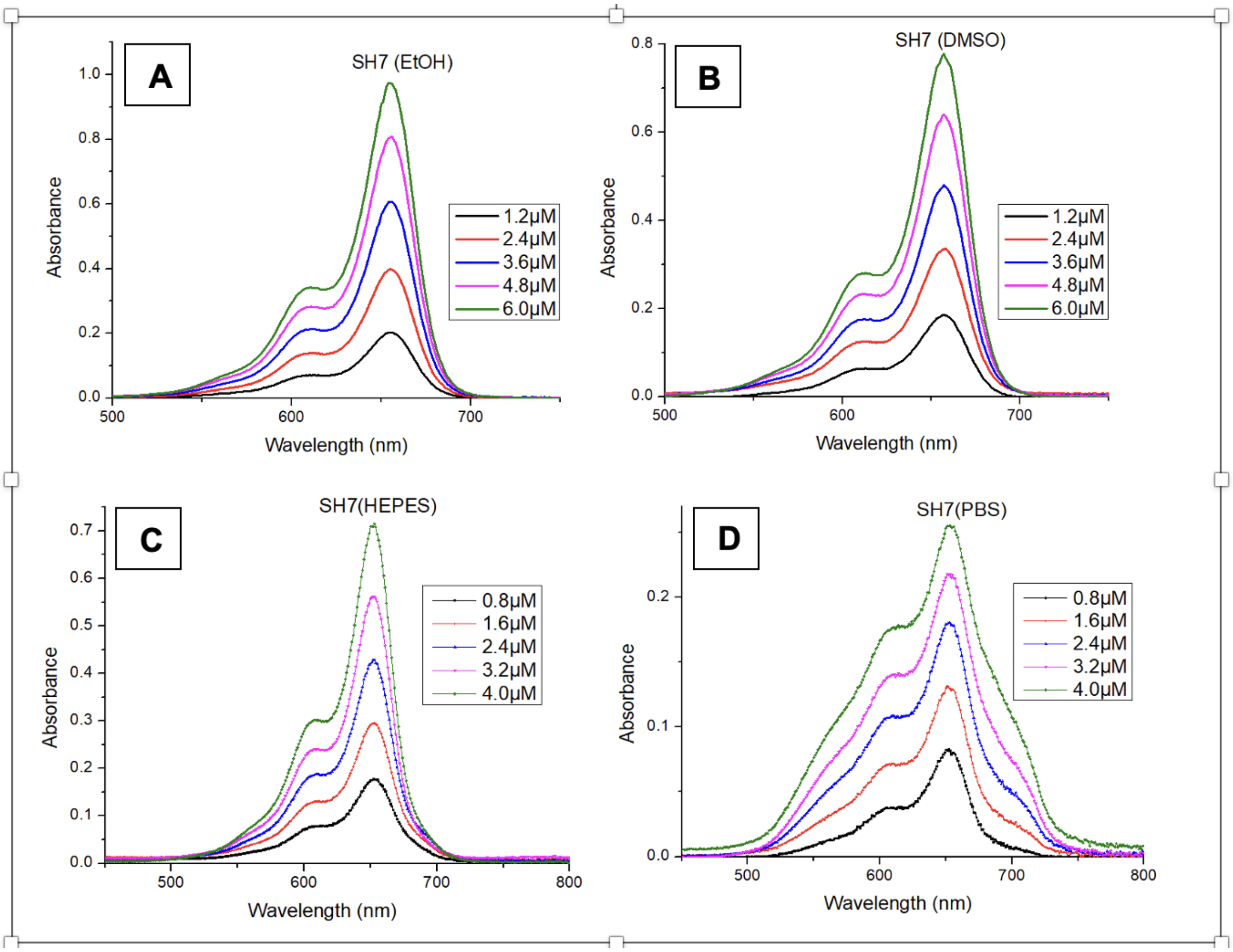

Figure S10. Concentrations dependent absorbances of fluorophore **27** in two organic solvents (EtOH, DMSO) and two buffer solutions (HEPES, PBS). The absorbance profiles of the **27** were recorded at various concentrations (0.8 µM, 1.6 µM, 2.4 µM, 3.2 µM and 4.0 µM) and the absorbance values obtained were plotted against wavelengths. Four media were used (A) EtOH, (B) DMSO, (C) HEPES, and (D) PBS.

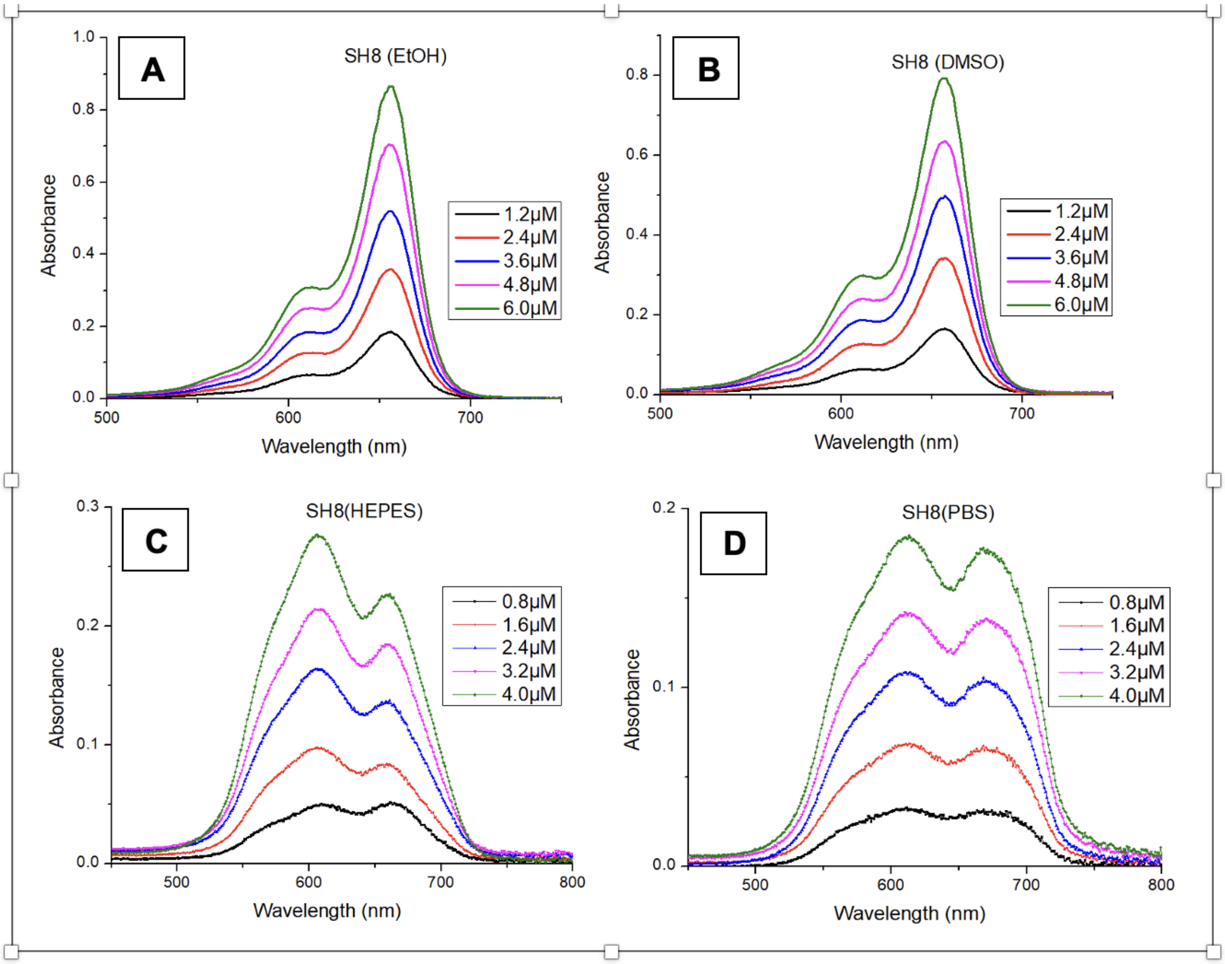

Figure S11. Concentrations dependent absorbances of fluorophore **28** in two organic solvents (EtOH, DMSO) and two buffer solutions (HEPES, PBS). The absorbance profiles of the **28** were recorded at various concentrations (0.8 µM, 1.6 µM, 2.4 µM, 3.2 µM and 4.0 µM) and the absorbance values obtained were plotted against wavelengths. Four media were used (A) EtOH, (B) DMSO, (C) HEPES, and (D) PBS.

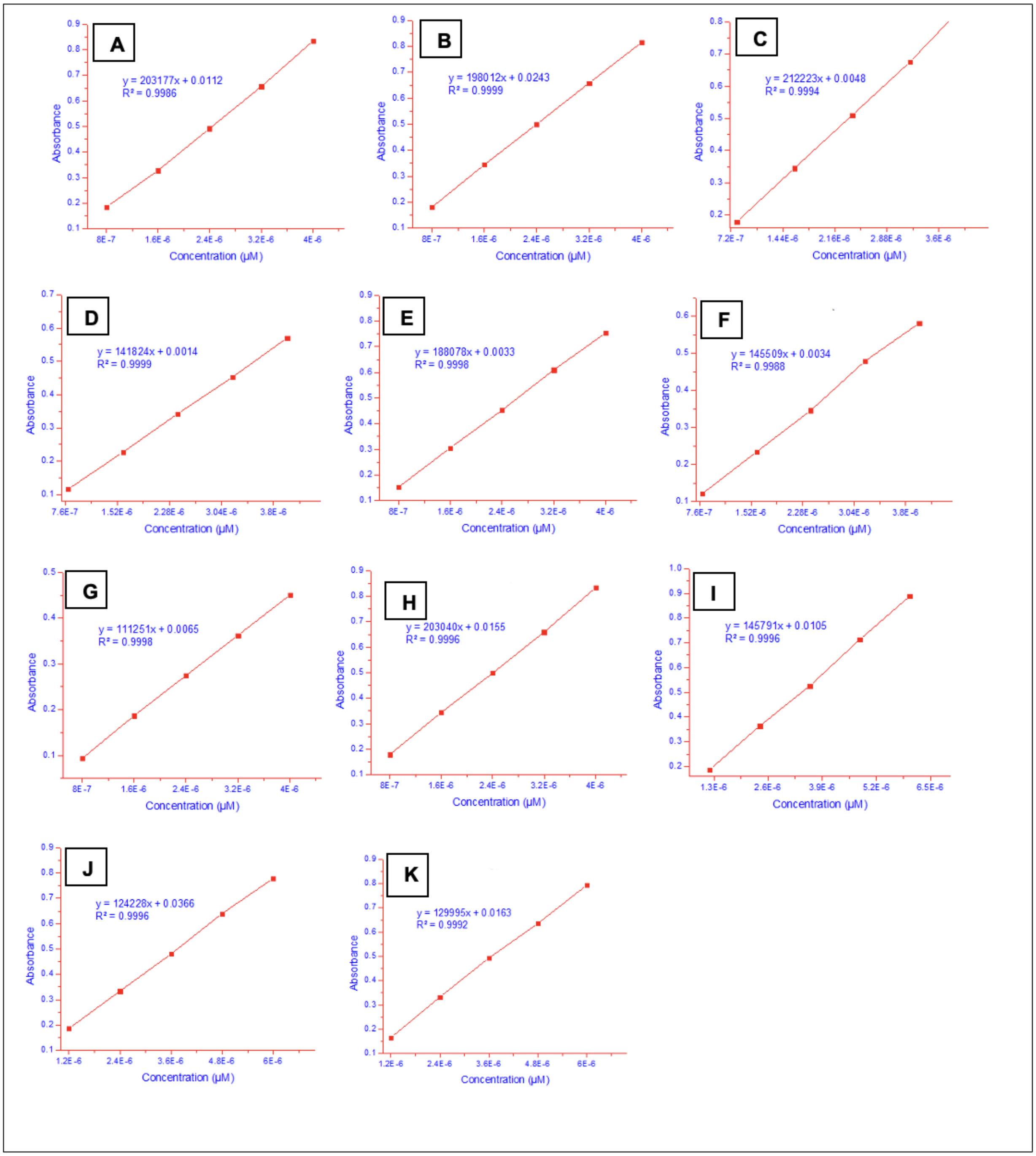

Figure S12. Molar extinction Coefficients **ε** ($M^{-1}cm^{-1}$) in DMSO (A) **18,** (B) **22**, (C) **20**, (D) **21**, (E) **19,** (F) **23**, (G) **24**, (H) **25,** (I) **26,** (J) **27** and (K) **28**.

Table S13. Percentage fluorescence intensities change with time of the selected fluorophores **18, 19, 20, 24**, **25** and **28** compared against **ICG.**

Time	28	18	20	19	25	24	ICG
**0**	100	100	100	100	100	100	100
**20**	100	99	99	98	100	96	93
**40**	99	99	97	95	97	92	86
**60**	99	99	97	94	94	88	80
**80**	99	98	95	92	91	83	73
**100**	100	98	94	91	86	80	67
**120**	100	96	93	88	84	78	59

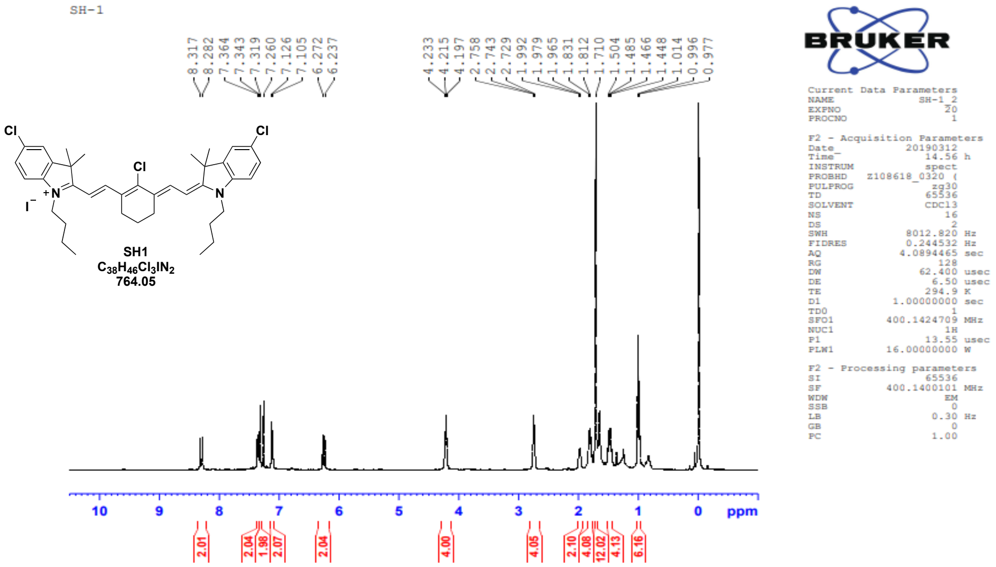

Figure S14. ^{1}H NMR spectrum of fluorophore **18**.

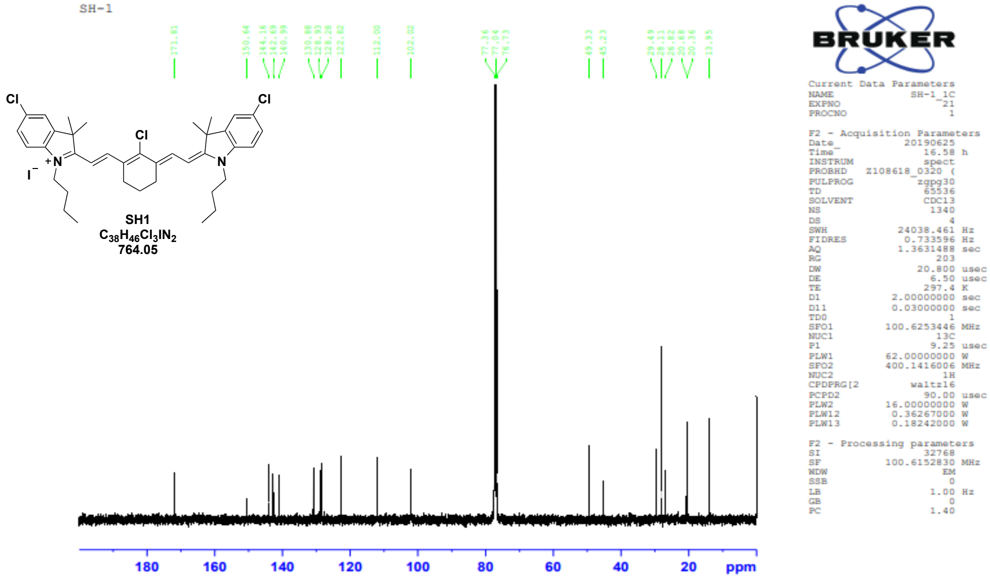

Figure S15. ^{13}C NMR spectrum of fluorophore **18**.

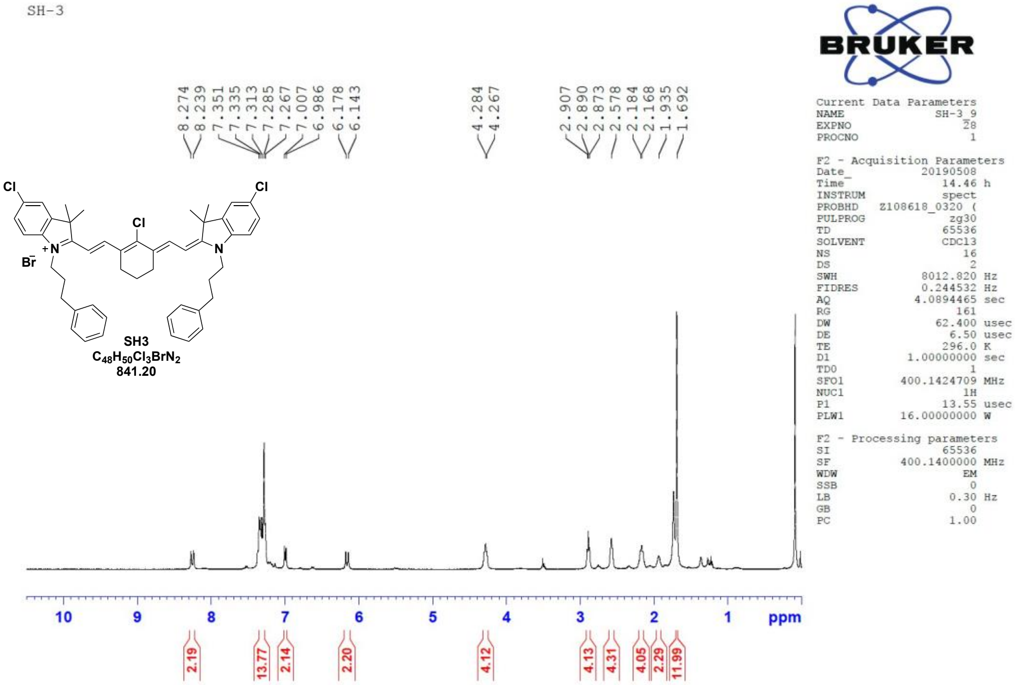

Figure S16. ^{1}H NMR spectrum of fluorophore **19**.

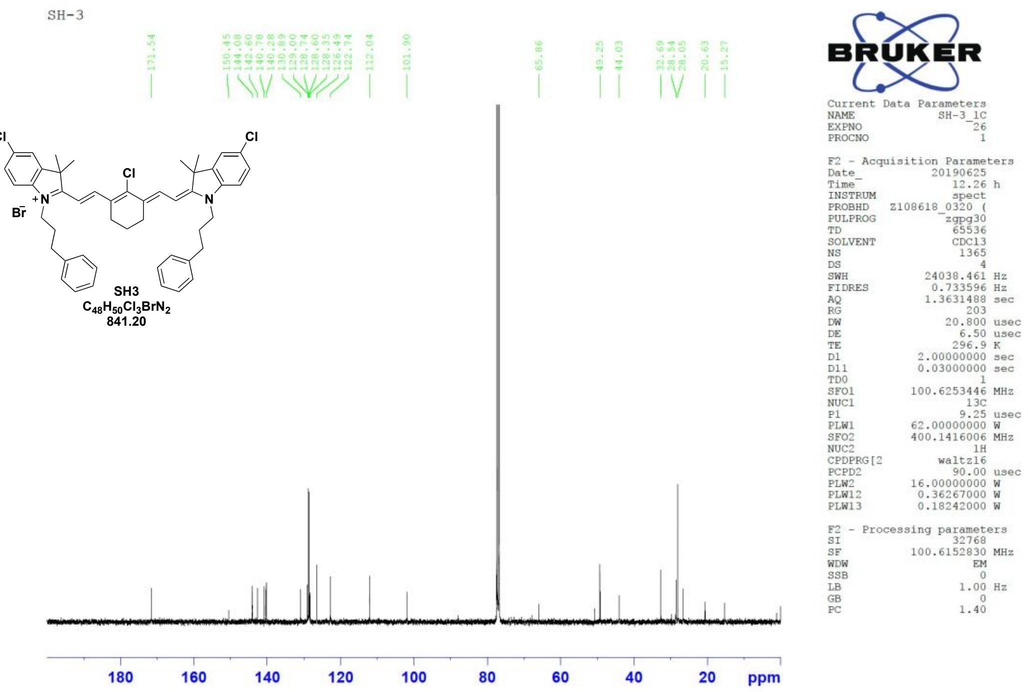

Figure S17. ^{13}C NMR spectrum of fluorophore **19**.

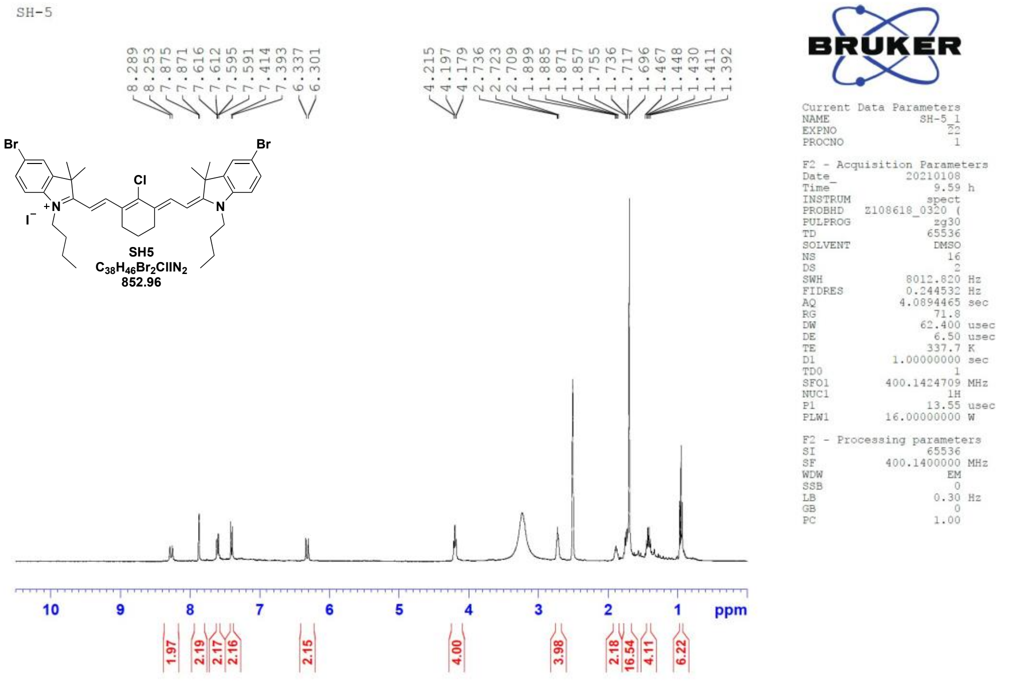

Figure S18. ^{1}H NMR spectrum of fluorophore **20**.

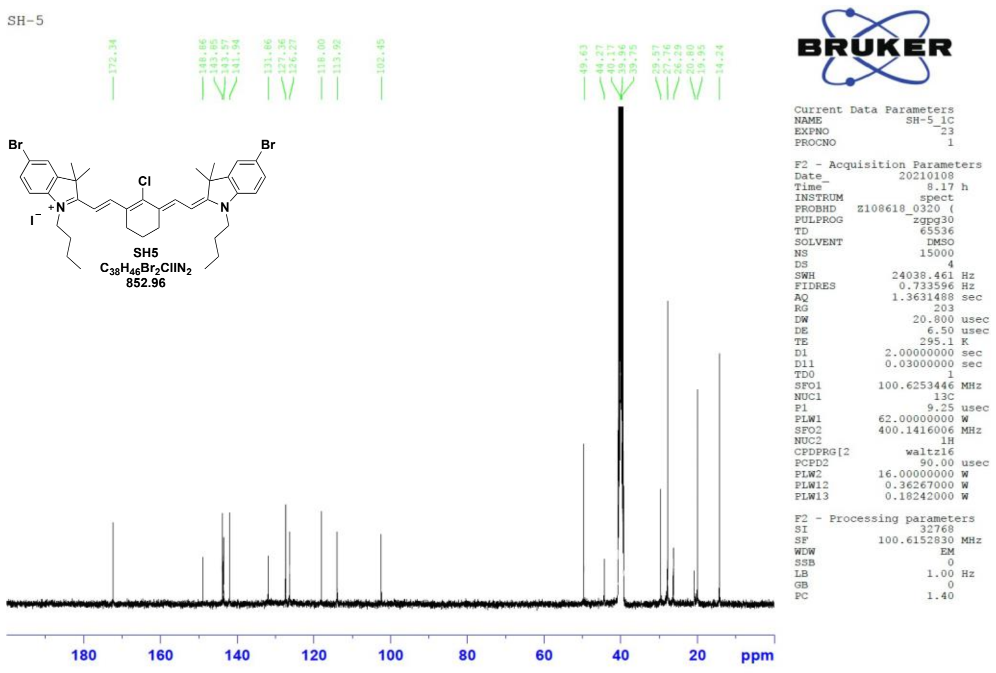

Figure S19. ^{13}C NMR spectrum of fluorophore **20**.

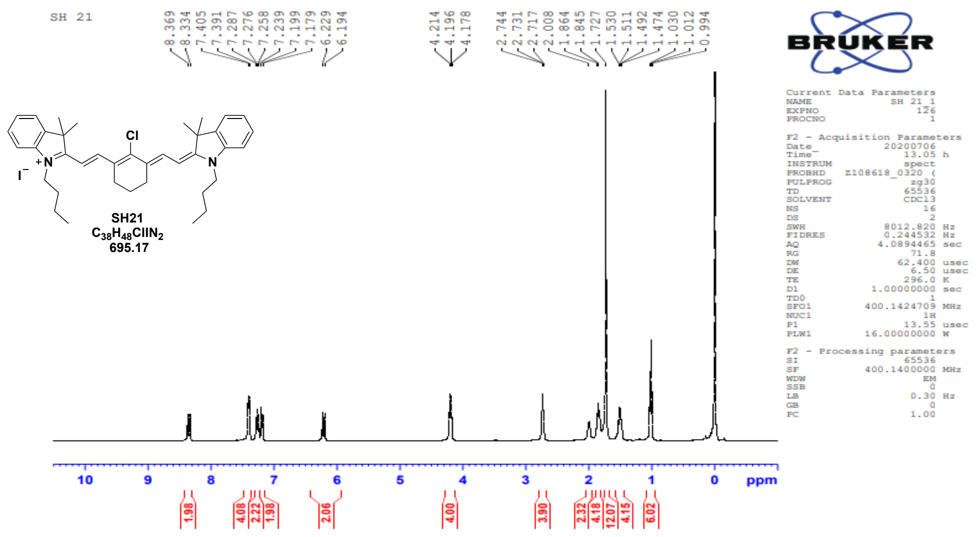

Figure S20. ^{1}H NMR spectrum of fluorophore **21**.

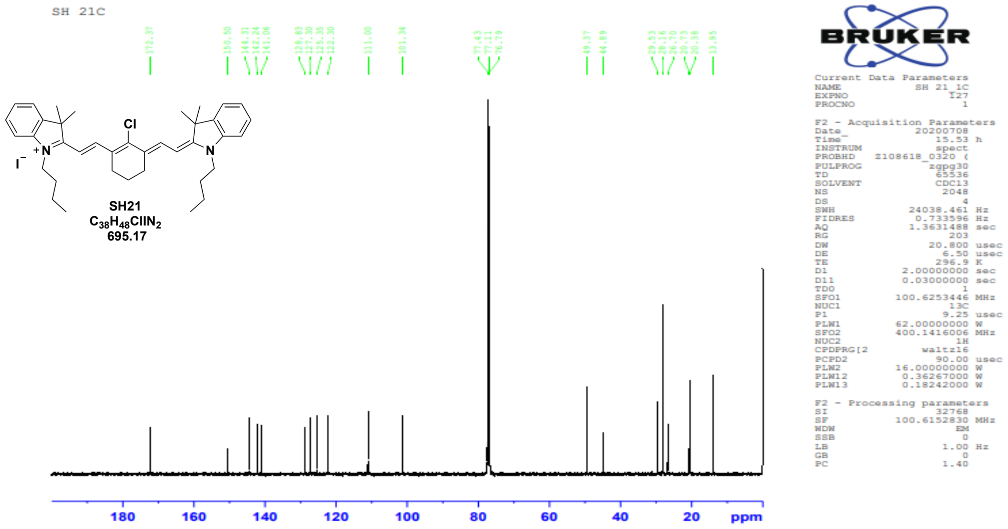

Figure S21. ^{13}C NMR spectrum of fluorophore **21**.

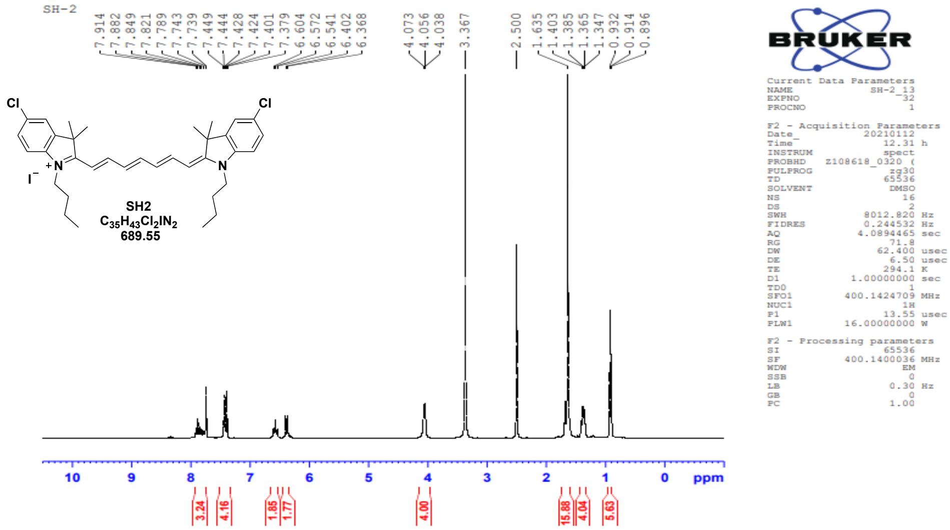

Figure S22. ^{1}H NMR spectrum of fluorophore **22**.

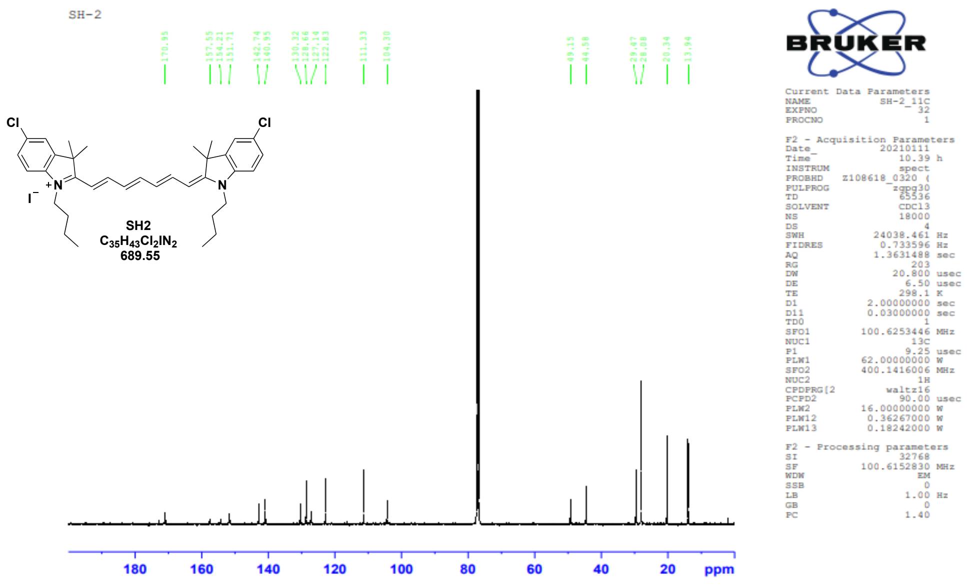

Figure S23. ^{13}C NMR spectrum of fluorophore **22**.

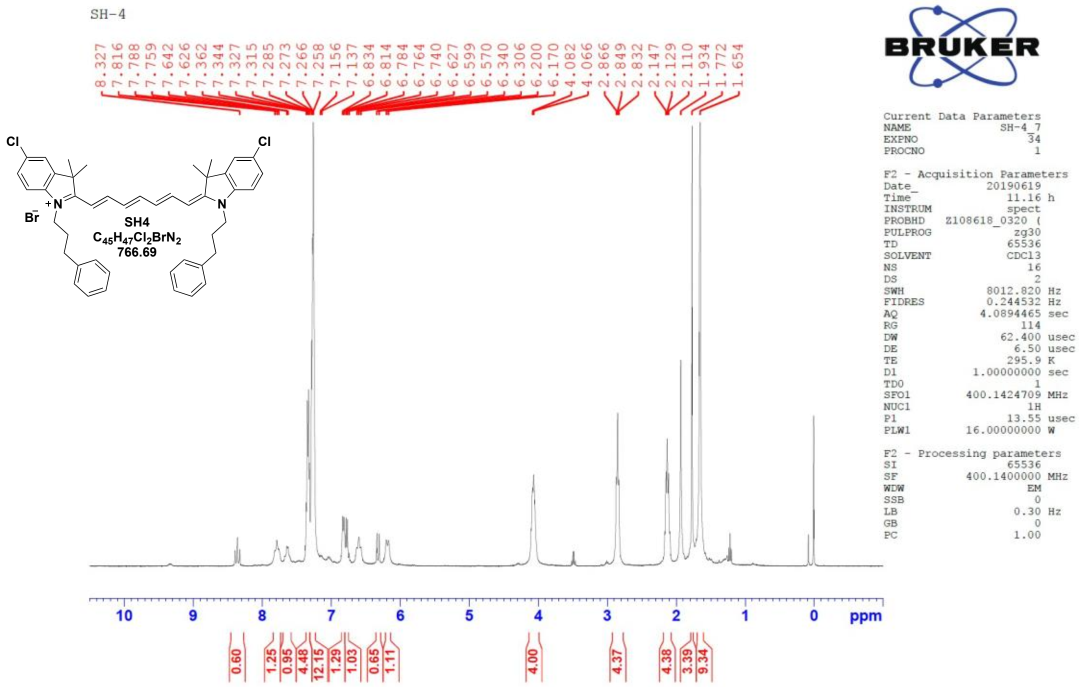

Figure S24. ^{1}H NMR spectrum of fluorophore **23**.

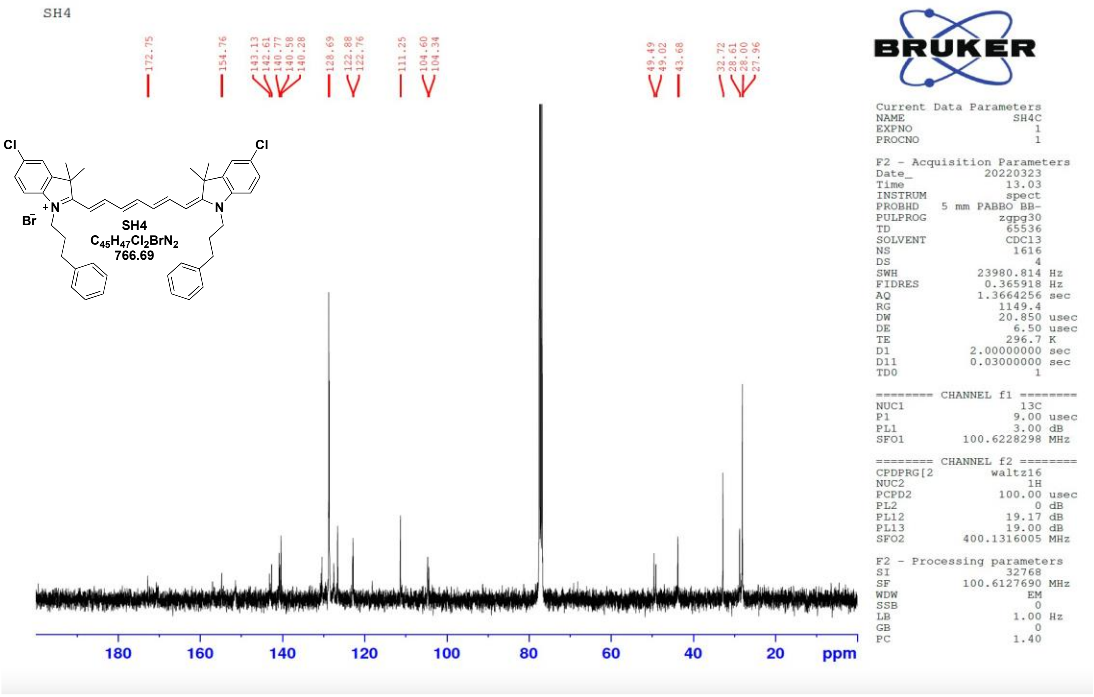

Figure S25. ^{13}C NMR spectrum of fluorophore **23**.

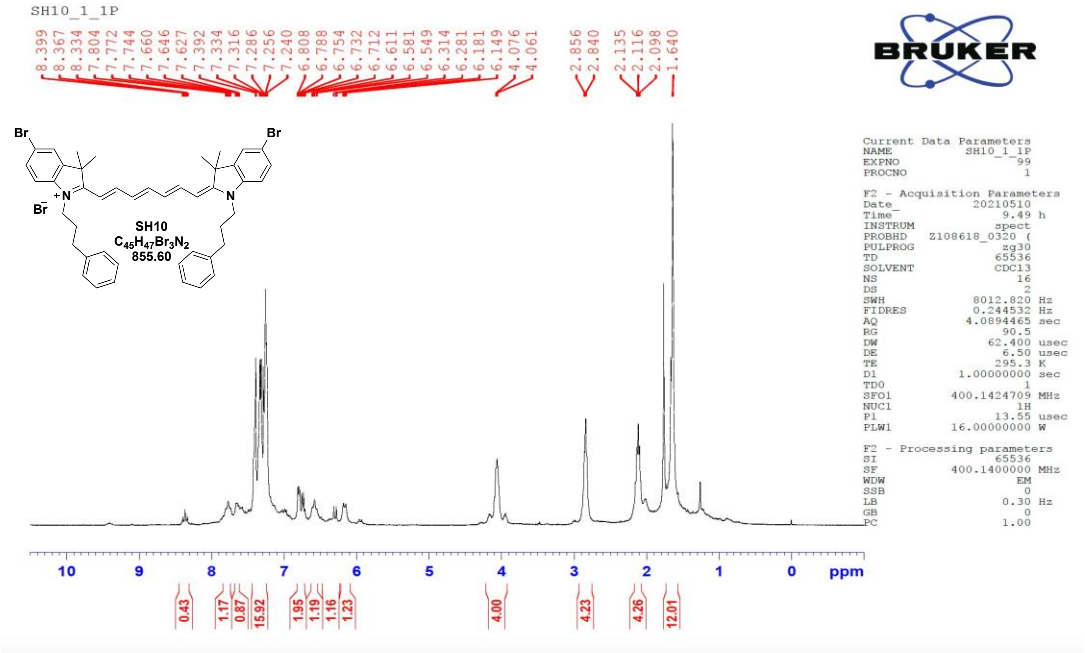

Figure S26. ^{1}H NMR spectrum of fluorophore **24**.

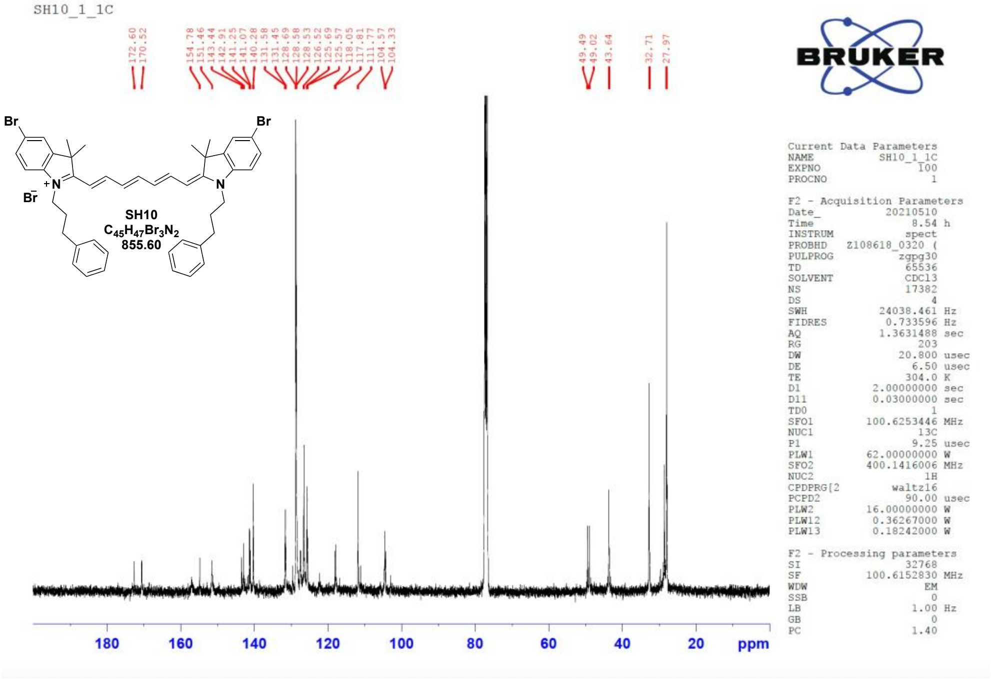

Figure S27. ^{13}C NMR spectrum of fluorophore **24**.

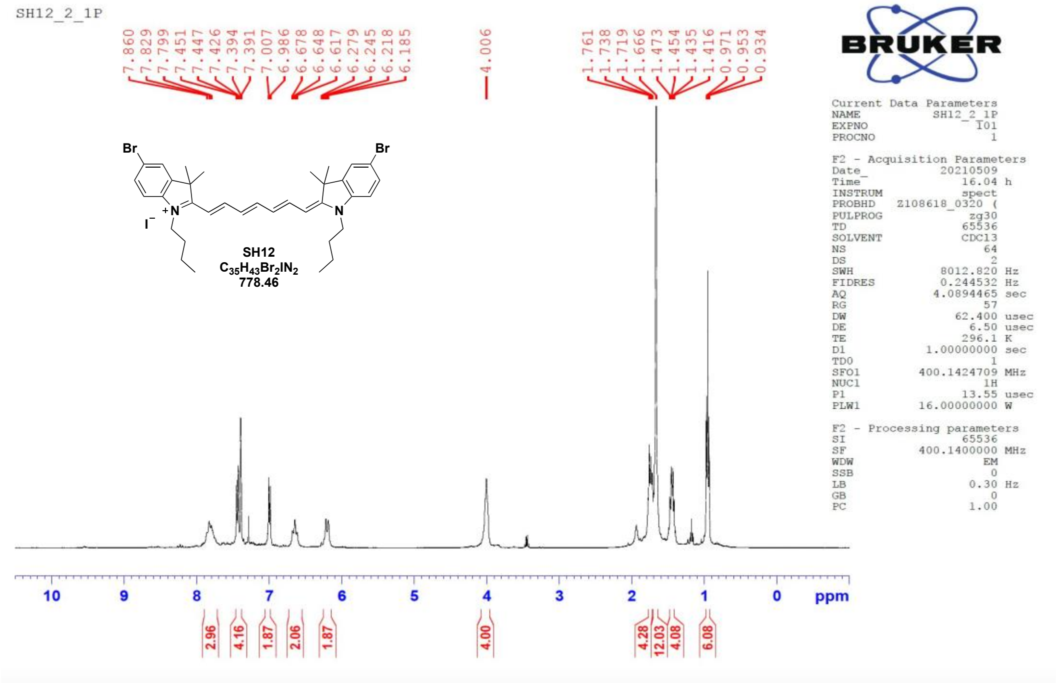

Figure S28. ^{1}H NMR spectrum of fluorophore **25**.

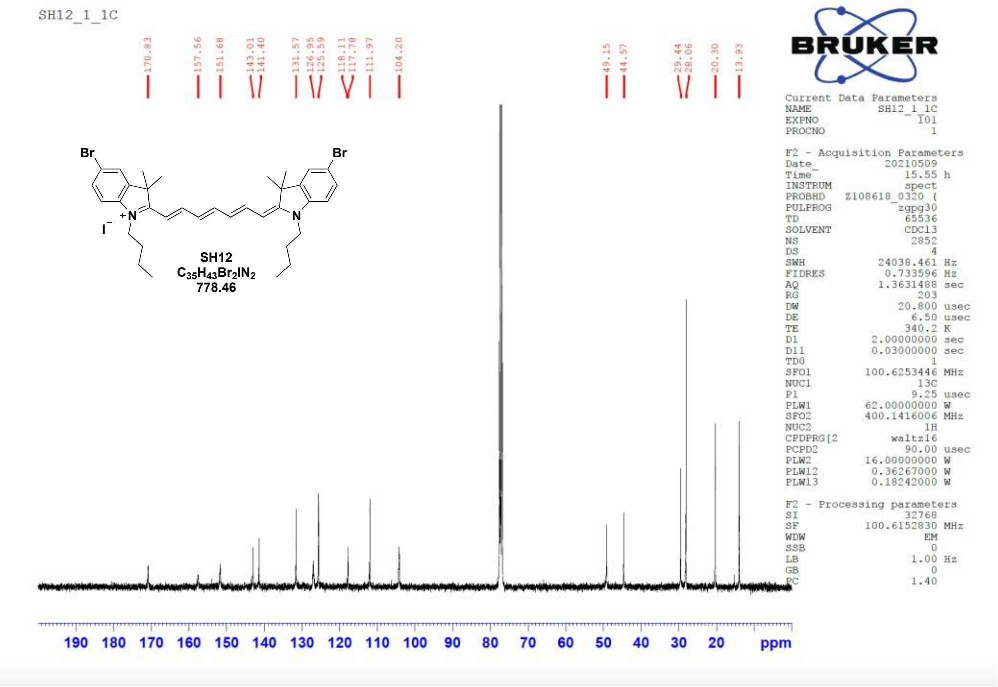

Figure S29. ^{13}C NMR spectrum of fluorophore **25**.

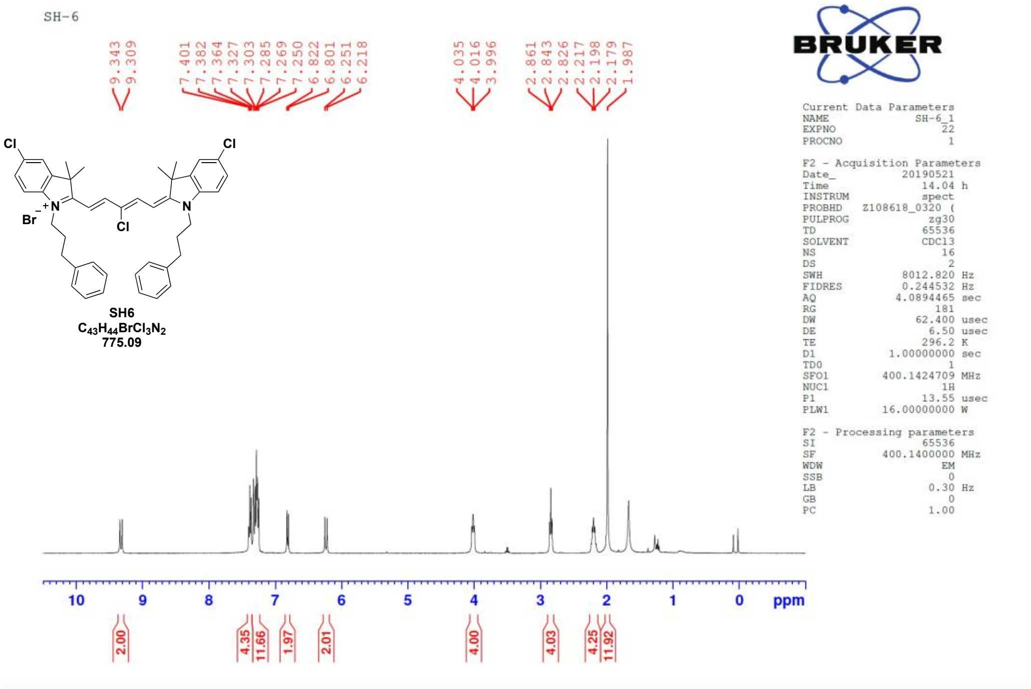

Figure S30. ^{1}H NMR spectrum of fluorophore **26**.

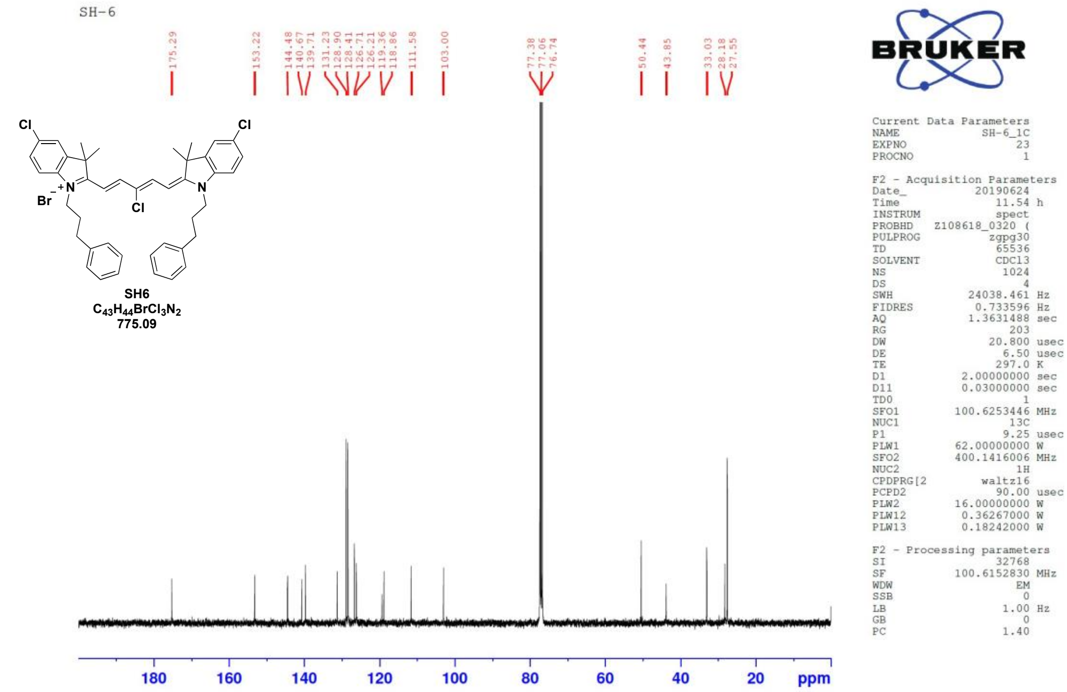

Figure S31. ^{13}C NMR spectrum of fluorophore **26**.

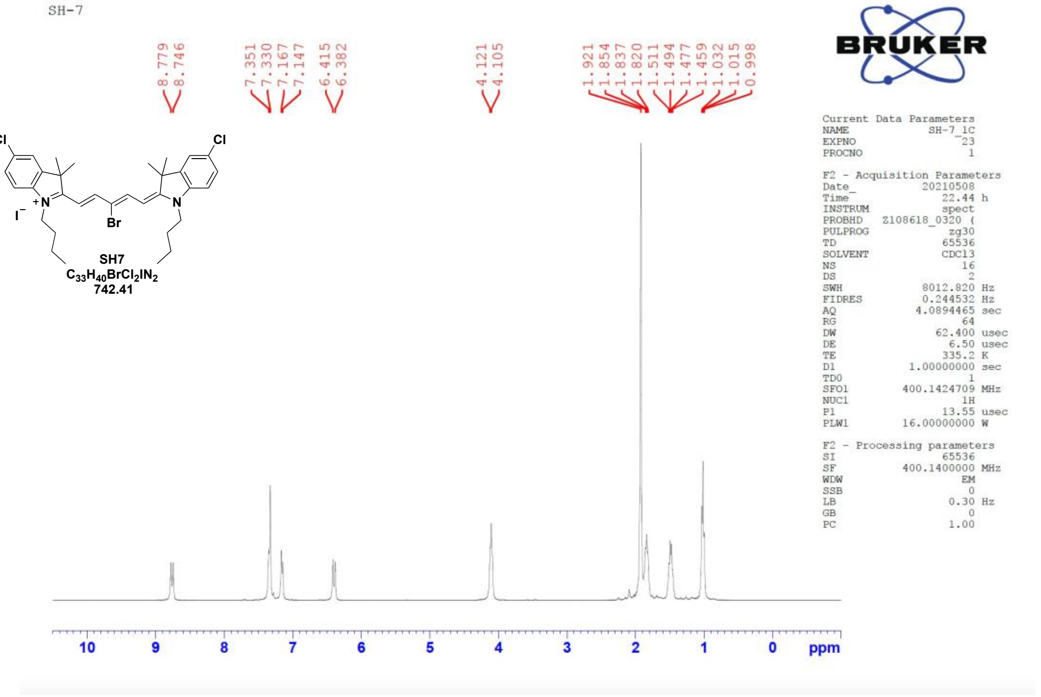

Figure S32. ^{1}H NMR spectrum of fluorophore **27**.

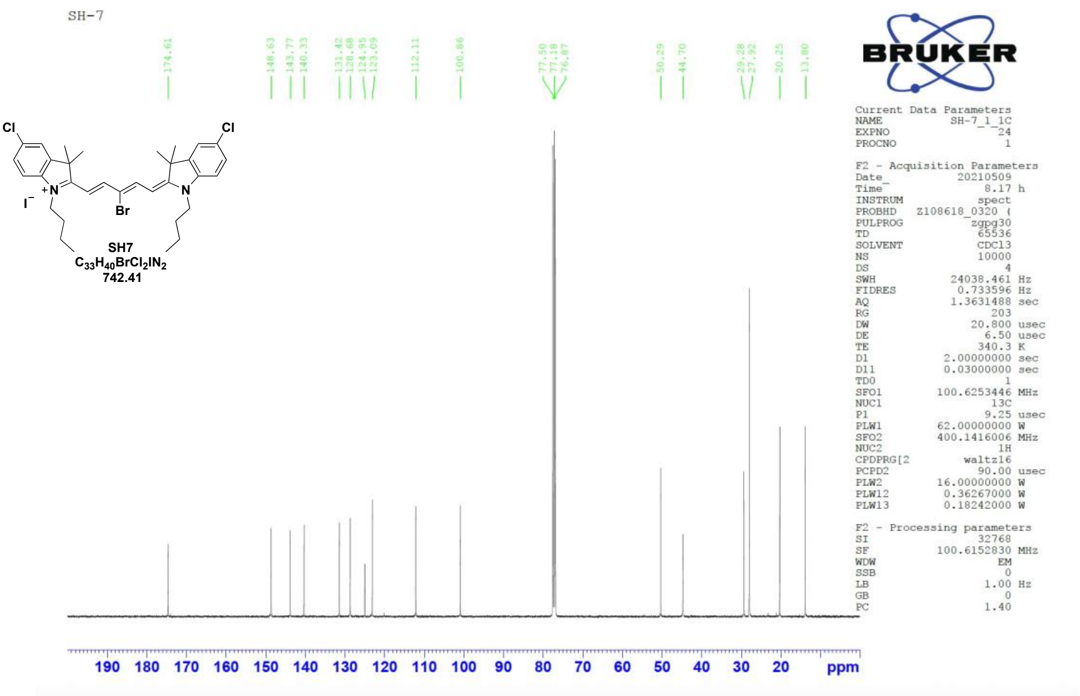

Figure S33. ^{13}C NMR spectrum of fluorophore **27**.

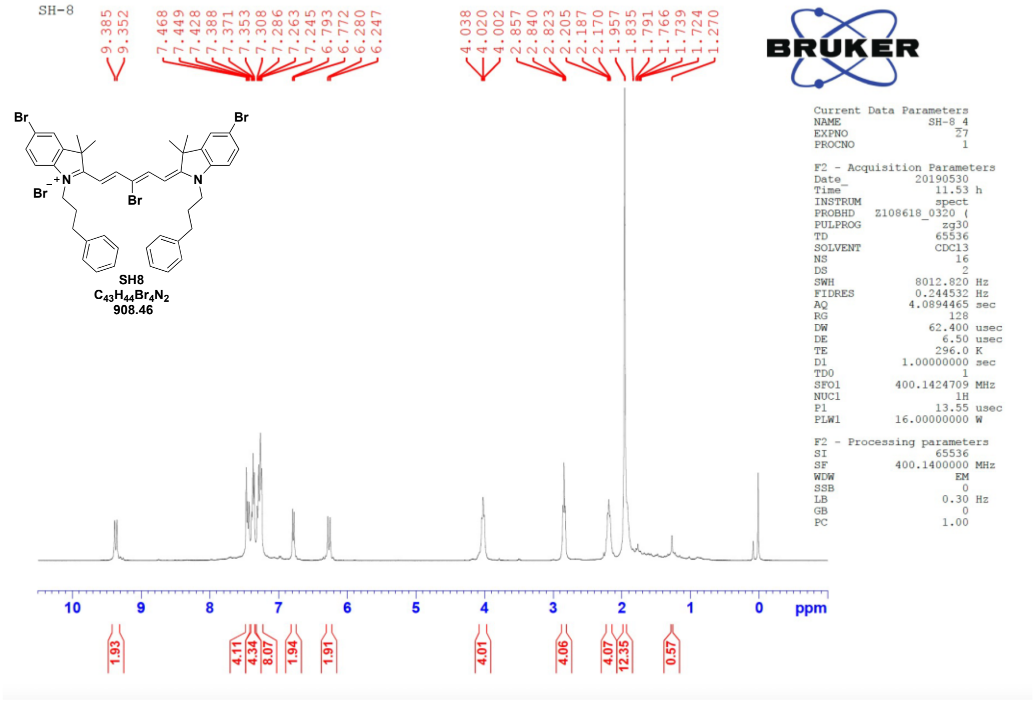

Figure S34. ^{1}H NMR spectrum of fluorophore **28**.

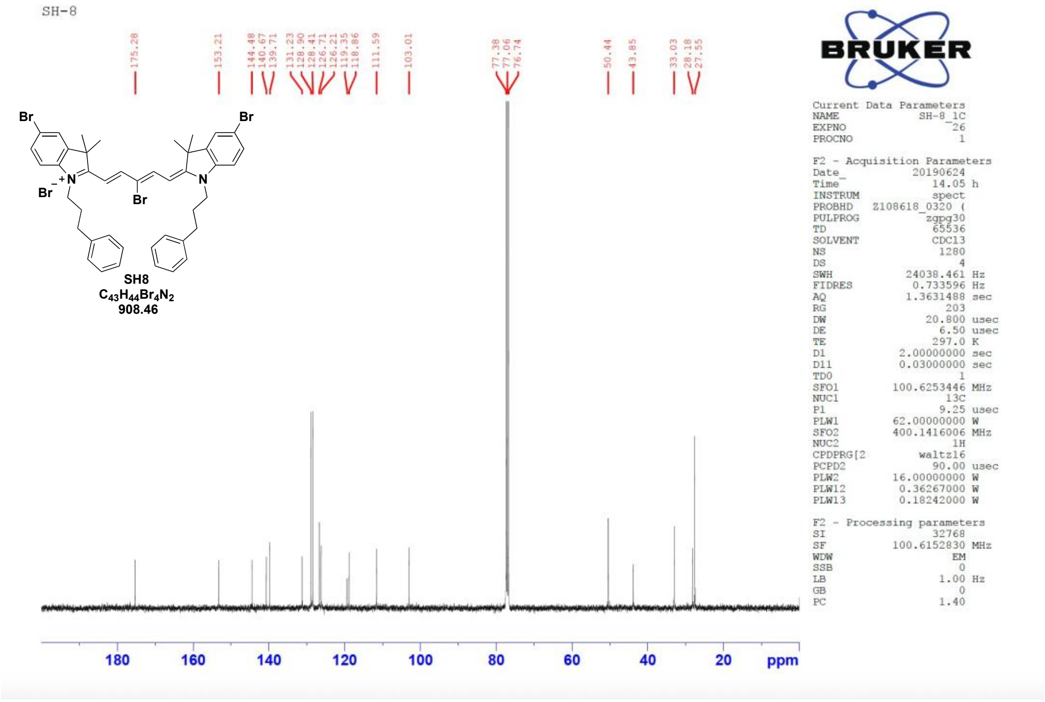

Figure S35. ^{13}C NMR spectrum of fluorophore **28**.
